# WDHD1 is essential for the survival of PTEN-inactive triple-negative breast cancer

**DOI:** 10.1038/s41419-020-03210-5

**Published:** 2020-11-21

**Authors:** Ayse Ertay, Huiquan Liu, Dian Liu, Ping Peng, Charlotte Hill, Hua Xiong, David Hancock, Xianglin Yuan, Marcin R. Przewloka, Mark Coldwell, Michael Howell, Paul Skipp, Rob M. Ewing, Julian Downward, Yihua Wang

**Affiliations:** 1 Biological Sciences, Faculty of Environmental and Life Sciences, University of Southampton, Southampton, SO17 1BJ UK; 2 Department of Oncology, Tongji Hospital, Tongji Medical College, Huazhong University of Science and Technology, 430030 Wuhan, China; 3 Oncogene Biology, The Francis Crick Institute, London, NW1 1AT UK; 4 Institute for Life Sciences, University of Southampton, Southampton, SO17 1BJ UK; 5 High-Throughput Screening, The Francis Crick Institute, London, NW1 1AT UK; 6 Centre for Proteomic Research, Institute for Life Sciences, University of Southampton, Southampton, SO17 1BJ UK; 7 NIHR Southampton Biomedical Research Centre, University Hospital Southampton, Southampton, SO16 6YD UK

**Keywords:** Cell signalling, Breast cancer

## Abstract

Triple-negative breast cancer (TNBC) is the most aggressive type of breast cancer that lacks the oestrogen receptor, progesterone receptor and human epidermal growth factor receptor 2, making it difficult to target therapeutically. Targeting synthetic lethality is an alternative approach for cancer treatment. TNBC shows frequent loss of phosphatase and tensin homologue (PTEN) expression, which is associated with poor prognosis and treatment response. To identify PTEN synthetic lethal interactions, TCGA analysis coupled with a whole-genome siRNA screen in isogenic PTEN-negative and -positive cells were performed. Among the candidate genes essential for the survival of PTEN-inactive TNBC cells, *WDHD1* (WD repeat and high-mobility group box DNA-binding protein 1) expression was increased in the low vs. high *PTEN* TNBC samples. It was also the top hit in the siRNA screen and its knockdown significantly inhibited cell viability in PTEN-negative cells, which was further validated in 2D and 3D cultures. Mechanistically, WDHD1 is important to mediate a high demand of protein translation in PTEN-inactive TNBC. Finally, the importance of WDHD1 in TNBC was confirmed in patient samples obtained from the TCGA and tissue microarrays with clinic-pathological information. Taken together, as an essential gene for the survival of PTEN-inactive TNBC cells, *WDHD1* could be a potential biomarker or a therapeutic target for TNBC.

## Introduction

Breast cancer is the most common cancer type and the leading cause of cancer death in women worldwide^1^. Triple-negative breast cancer (TNBC) lacks the oestrogen receptor (ER), progesterone receptor (PR) and human epidermal growth factor receptor 2 (HER2), and accounts for between 10 and 20% of breast cancers^2–5^. TNBC is the most aggressive and high-grade breast cancer type with high risk of tumour recurrence and metastasis compared to the other breast cancer subtypes^6^. As TNBC lacks all three receptors, this causes more challenges for the treatment of the disease. Chemotherapy has been the only standard treatment option to improve the overall survival rate of TNBC patients for several years^7^. Therefore, it is important to study gene profiling by identifying different gene expression signatures in TNBC to discover a novel biomarker or targeted therapy for the disease. Atezolizumab (TECENTRIQ^®^), an anti-programmed death-ligand 1 (PD-L1) monoclonal antibody (checkpoint inhibitor), was approved as the first breast cancer immunotherapy to be combined with chemotherapy (Abraxane; nab®-Paclitaxel) for PD-L1-positive TNBC^8^. As a heterogeneous disease^9^, further gene profiling studies are required to identify novel biomarkers or therapeutic targets for TNBC.

TNBC shows frequent loss of phosphatase and tension homologue (PTEN) expression compared to the other molecular subtypes of breast cancer^10,11^. It has been shown that loss of PTEN expression was significantly associated with TNBC that shows poor prognosis and significant links with high-grade tumour, larger tumour size, lymph node metastasis and tumour recurrence^12^. PTEN was identified as a tumour suppressor gene (TSG), located on 10q23 chromosome band, which plays an essential role to control cell cycle, growth and survival^13^. Mechanistically, PTEN has a cytoplasmic lipid phosphatase role that can inhibit the phosphatidylinositol 3-kinase (PI3K)-AKT pathway^13,14^, and the nuclear phosphatase-independent role of PTEN which has been shown to maintain genomic stability^15,16^.

Targeting synthetic lethality is an alternative approach for cancer treatment^17^. To identify novel targeted therapies, synthetic lethality screens were performed, including RNA interference (RNAi) screens^18,19^. One of the well-known examples of synthetic lethality interaction is between *BRCA1*/*2* and *PARP*. *BRCA1*/*2* are TSGs that have a role in homologous-recombination-mediated DNA repair and *PARP* is involved in base excision repair. Tumours with *BRCA1*/*2* deficiency depend on *PARP1* for DNA repair. Thus, inhibition of PARP1 kills *BRCA1*/*2*-deficient tumours^20,21^. Discovering PTEN synthetic lethal interactions in TNBC may provide potential biomarkers or targeted therapies for this breast cancer type that does not have successful treatment options.

In this study, candidate genes essential for the survival of PTEN-inactive TNBC cells were identified by the TCGA analysis and a whole-genome siRNA screen in isogenic PTEN-negative and -positive cells. Among them, WD repeat and high-mobility group box DNA-binding protein 1 (*WDHD1*) expression was increased in the low vs. high *PTEN* TNBC samples. It was also the top candidate gene whose knockdown significantly inhibited cell viability in PTEN-negative cells, which was further validated in 2D and 3D cultures. Mechanistically, WDHD1 was important to mediate a high demand of protein translation in PTEN-inactive TNBC. Finally, the importance of WDHD1 in TNBC was confirmed in patient samples obtained from the TCGA and tissue microarrays with clinic-pathological information.

## Results

### TCGA analysis confirms PTEN expression is decreased in TNBC and correlates with clinical stages

It has been stated that PTEN inactivation occurs more frequently in TNBC than the other subtypes of breast cancer^11,12^. To confirm this finding, clinical data of breast invasive carcinoma (TCGA, PanCancer) was obtained from cBioportal (https://www.cbioportal.org/). *PTEN*, mRNA levels were analysed in the normal breast samples and each molecular subtypes of breast cancer. *PTEN*, mRNA levels were significantly lower in TNBC compared to the normal breast, luminal A, luminal B and HER2+ subtypes, although PTEN mutation frequency was similar (~6%) across all subtypes of breast cancer (Supplementary Fig. S1a; *P* < 0.0001).

Protein (RPPA) TCGA breast invasive carcinoma data from the UCSC Cancer Genome Browser (https://genome-cancer.ucsc.edu/) was obtained. The categorised TNBC samples (TCGA, Provisional) from the cBioportal website was aligned with protein (RPPA) data (see Supplementary Materials). A significant correlation between mRNA and protein levels of PTEN (Supplementary Fig. S1b; *r* = 0.55; *P* = 0.0001) suggested that PTEN inactivation in TNBC occurs at the transcriptional level. The number of patients with T2 and above, or Stage II and above, in PTEN high TNBC samples was significantly lower than the PTEN low group (Supplementary Fig. S1c, d; *P* < 0.05). Functionally, decreased PTEN levels were responsible for the high AKT activity in TNBC, since there was a significant negative correlation between the levels of phosphorylated AKT (AKT1_PT308, a main downstream molecule of PTEN^22^) and PTEN in TNBC (Supplementary Fig. S1e; *r* = −0.55; *P* = 0.0001).

These findings confirm that reduced PTEN levels correlate with advanced clinical stages and a high AKT activity in TNBC.

### Candidate genes essential for the survival of PTEN-inactive TNBC cells are identified by the TCGA analysis and a whole-genome siRNA screen

**Fig. 1:**
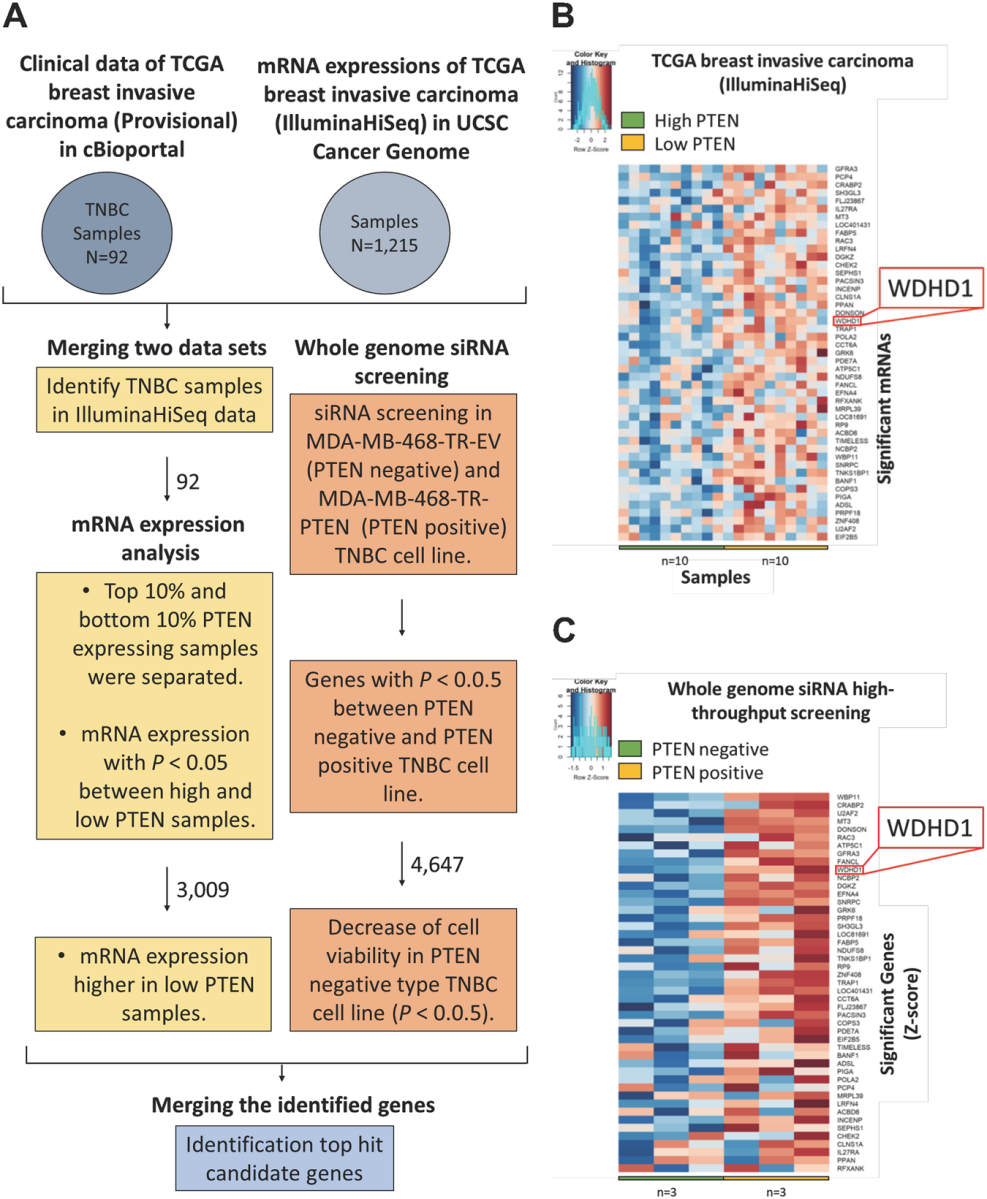
Candidate genes essential for the survival of PTEN-inactive TNBC cells are identified by the TCGA analysis and a whole-genome siRNA screen. **A** Workflow showing the analysis to identify 47 candidate genes essential for the survival of PTEN-inactive TNBC cells. **B** Heatmap showing 47 candidate mRNAs that are over-expressed in TNBC samples with the low PTEN compared to those with the high PTEN from TCGA analysis. Red indicates up-regulation and blue for down-regulation. *n* = 10 per group. **C** Heatmap showing 47 candidate genes that are required for the survival of PTEN-negative TNBC cells from a whole-genome siRNA screen. Red indicates high *Z* scores and blue for low *Z* scores. *n* = 3 per group.

As shown in Fig. 1A, 92 TNBC samples were identified from TCGA. *PTEN*, mRNA expression was widely distributed across all TNBC samples; therefore, the top 10% and bottom 10% of samples were defined as high and low *PTEN*, respectively. In all, 3009 mRNAs were identified as differentially expressed in the high vs. low *PTEN* groups (Supplementary Fig. S1f; *P* < 0.05).

A whole-genome siRNA screen was performed in isogenic GFP-labelled PTEN-negative (PTEN−) cells and CherryFP-labelled PTEN-positive (PTEN+) cells (Supplementary Figs. S2 and S3a; details in Supplementary Materials). In all, 4647 genes were identified as showing differential effects on cell viability in PTEN− vs. PTEN+ cells (Supplementary Fig. S3b; *P* < 0.05).

By cross-referencing TCGA analysis with the whole-genome siRNA screen, 47 candidate genes essential for the survival of PTEN-inactive TNBC cells were identified (Fig. 1B, C and Supplementary Tables S1 and S2). Among them, *WDHD1* expression was increased in the low vs. high *PTEN* TNBC samples (Supplementary Table S1; *P* = 0.03). It was also the top candidate gene whose knockdown significantly inhibited cell viability in PTEN-negative cells (*Z* score = −1.26) with mild effects on PTEN-positive cells (Supplementary Table S2; *Z* score = −0.32; *P* = 0.009).

### WDHD1 expression is affected by PTEN status in TNBC cells

**Fig. 2:**
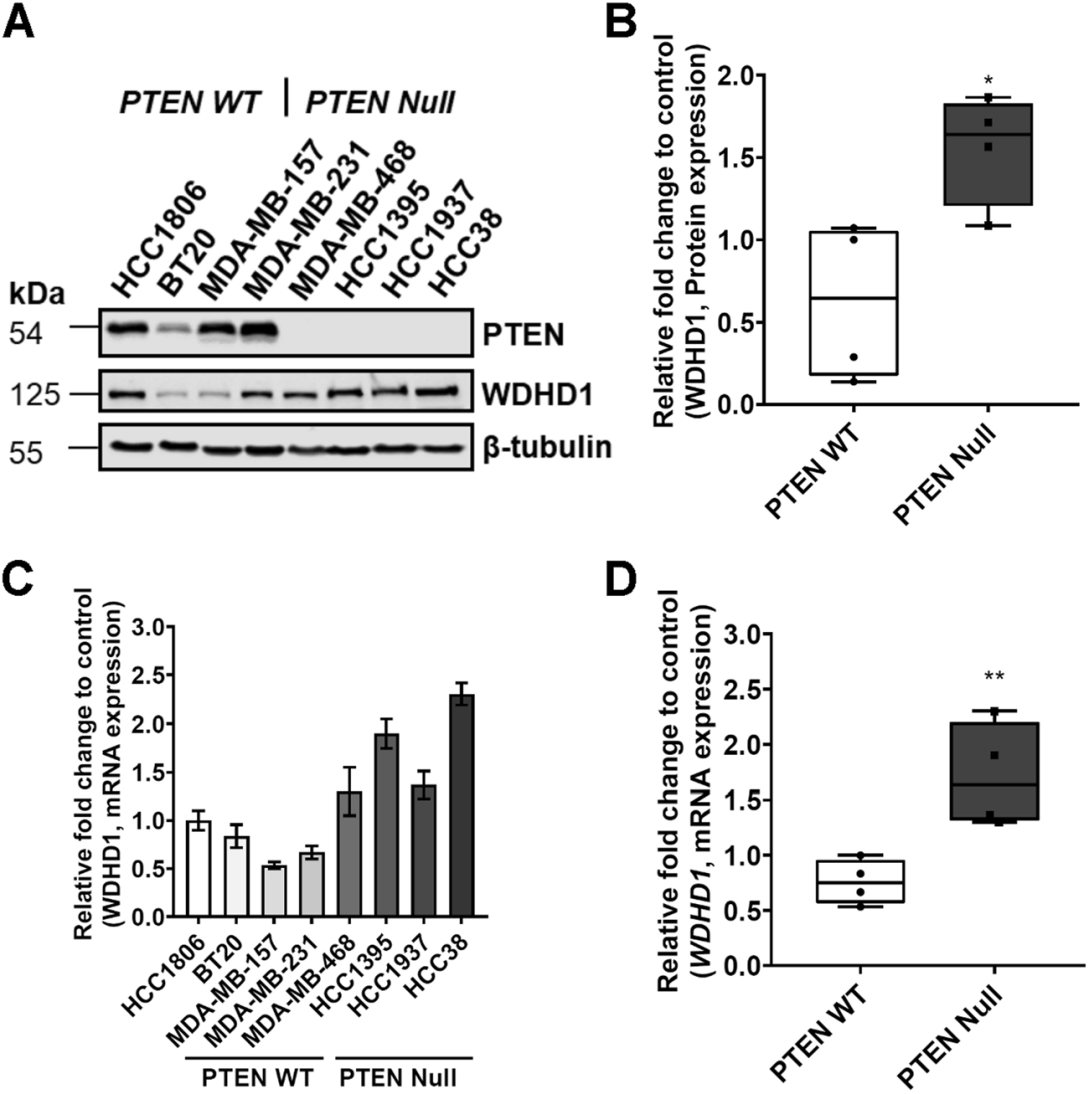
WDHD1 is highly expressed in PTEN-inactive TNBC cells. **A** Protein expression of WDHD1 and PTEN in the indicated TNBC cell lines with PTEN WT (wild-type) or PTEN null. β-tubulin was used as a loading control. **B** Graph showing protein levels of WDHD1 in PTEN WT or PTEN null TNBC cell lines. *P < 0.05. **C** Fold change in mRNA levels of *WDHD1* in the indicated PTEN WT or PTEN null TNBC cell lines. *WDHD1* mRNA expression was normalised to a housekeeping gene, β-actin. Data are mean ± SEM. *n* = 3. **D** Graph showing mRNA levels of *WDHD1* in PTEN WT or PTEN null TNBC cell lines. **P < 0.01. Data in (**B**) and (**D**) are individual values with mean, and error bars indicate minimum and maximum individual values. *n* = 4 per group.

TCGA analysis suggested that *WDHD1* expression is increased in the low vs. high *PTEN* TNBC samples. To validate this finding, both protein and mRNA levels of WDHD1 were measured in a panel of TNBC cell lines, either PTEN WT (HCC1806, BT20, MDA-MB-157 and MDA-MB-231) or PTEN null (MDA-MB-468, HCC1395, HCC1937 and HCC38). We found WDHD1 was highly expressed at both the protein (Fig. 2A, B; *P* < 0.05) and mRNA (Fig. 2C, D; *P* < 0.01) level in PTEN null vs. WT TNBC cell lines.

**Fig. 3:**
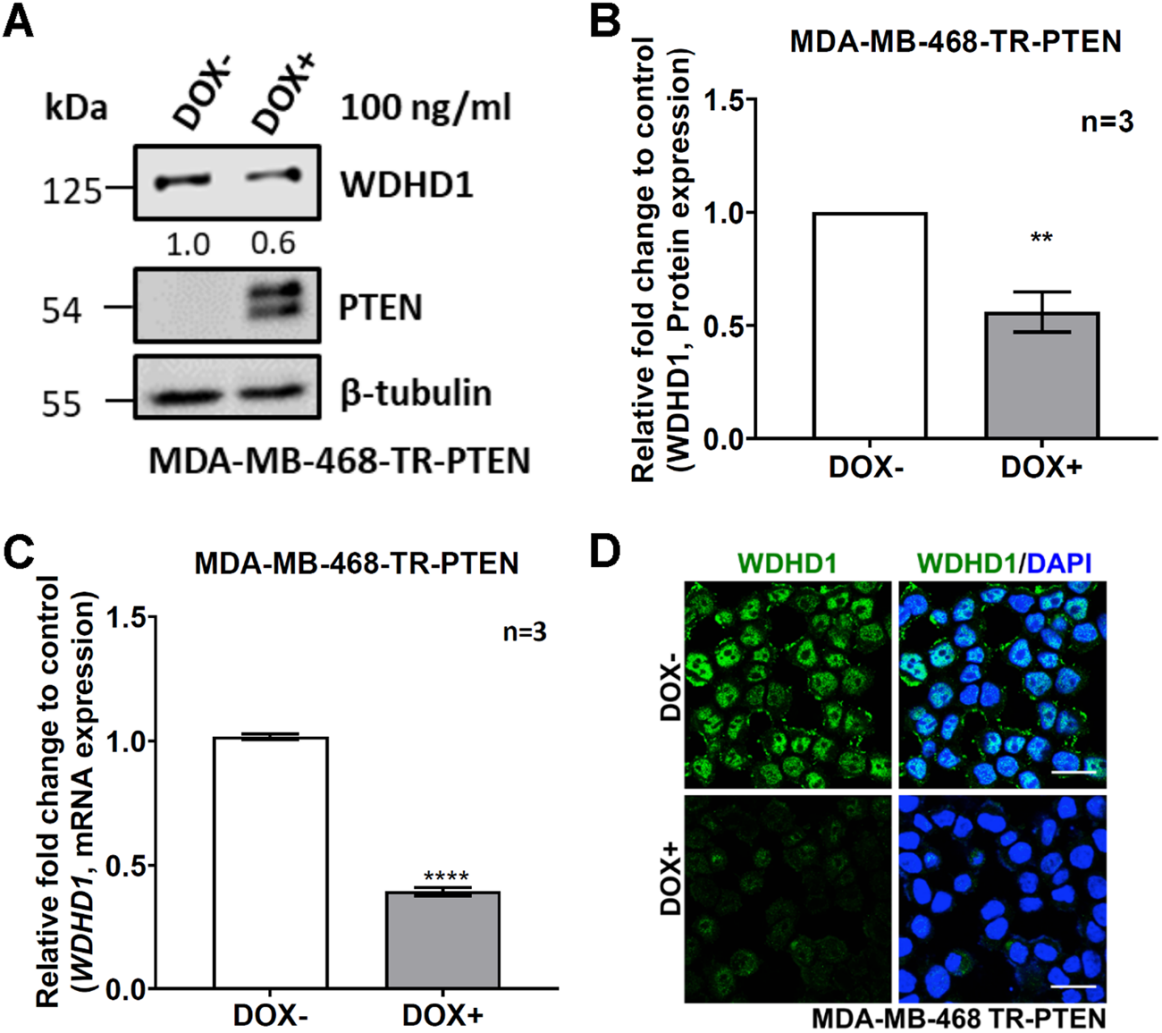
WDHD1 levels are reduced upon PTEN expression in MDA-MB-468 cells. **A** Protein expression of PTEN and WDHD1 in MDA-MB-468-TR-PTEN cells treated with or without doxycycline (DOX). β-tubulin was used as a loading control. Adding DOX induces PTEN expression in MDA-MB-468-TR-PTEN cells. Graphs showing protein (**B**) or mRNA (**C**) levels of WDHD1 in MDA-MB-468-TR-PTEN cells treated with (DOX+) or without DOX (DOX−). **P < 0.01. ****P < 0.0001. Data are mean ± SEM. *n* = 3 per group. **D** Immunofluorescence staining of WDHD1 (green) in MDA-MB-468-TR-PTEN cells treated with (DOX+) or without DOX (DOX−). 4'6-Diamidino-2-Pheylindole (DAPI) (blue) was used to stain nuclei. Scale bars: 20 μm.

To further confirm the relationship between PTEN and WDHD1 expression levels, we introduced into MDA-MB-468 cells (PTEN null) a regulatable PTEN construct that is conditionally responsive to doxycycline (DOX). Addition of DOX induces PTEN expression in MDA-MB-468 cells expressing TR-PTEN (MDA-MB-468-TR-PTEN) to a similar level in a non-tumorigenic triple-negative human breast epithelial cell line MCF10A (Supplementary Fig. S2a). As shown in Fig. 3, WDHD1 levels were significantly reduced upon PTEN expression (DOX+) in MDA-MB-468-TR-PTEN cells at both mRNA and protein levels, as demonstrated by the results from the western blot (Fig. 3A, B; *P* < 0.01), qRT-PCR (Fig. 3C; *P* < 0.0001) and immunofluorescence staining of WDHD1 (Fig. 3D).

**Fig. 4:**
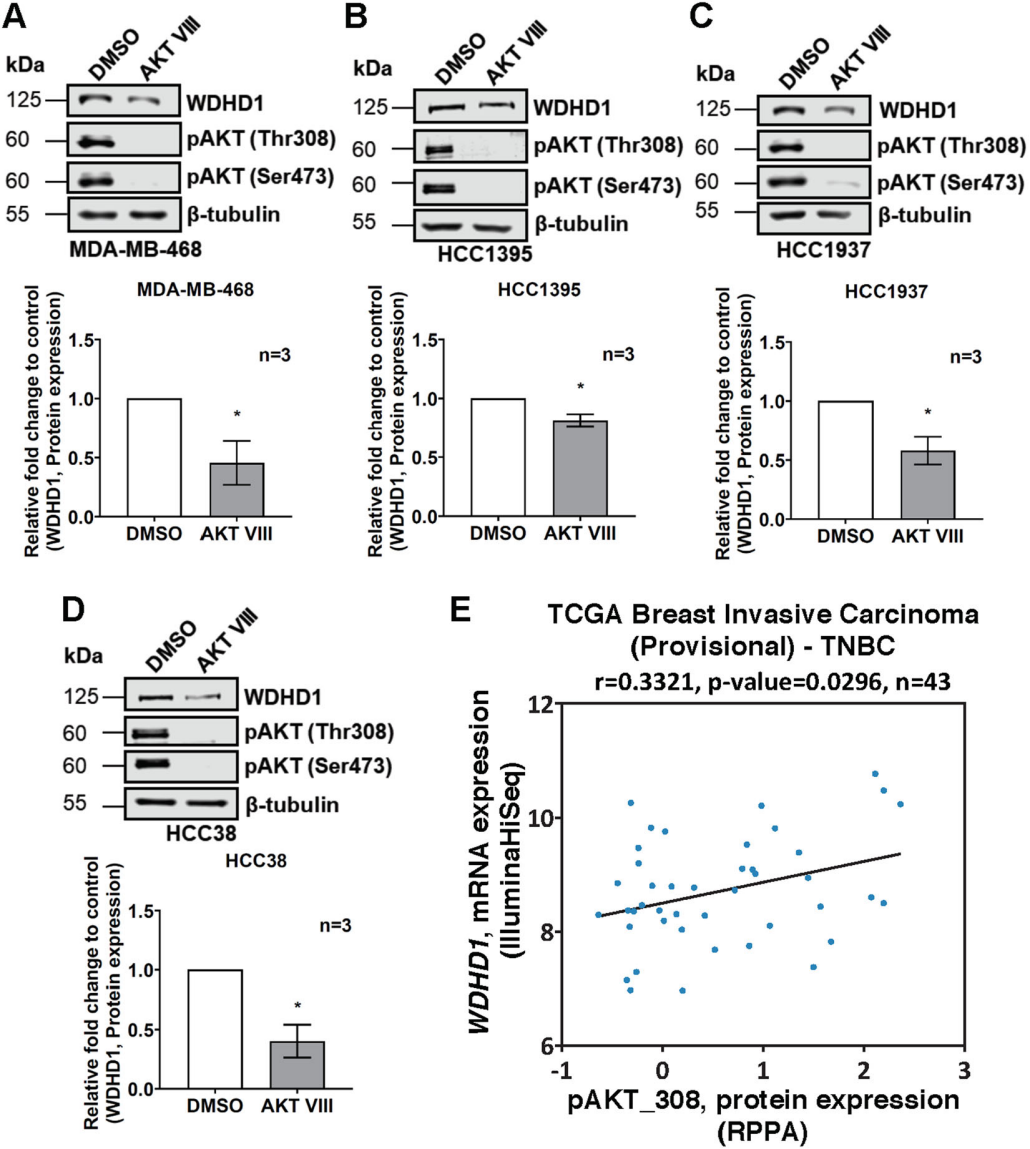
WDHD1 levels are reduced upon AKT inhibition in PTEN null TNBC cells. Protein expression of WDHD1, phospho-AKT (pAKT) (Thr308) and pAKT (Ser473) in MDA-MB-468 (**A**), HCC1395 (**B**), HCC1937 (**C**) and HCC38 (**D**) treated with DMSO or an AKT inhibitor, AKT VIII (10 μM). β-tubulin was used as a loading control. Graphs showing protein levels of WDHD1 in MDA-MB-468 (**A**), HCC1395 (**B**), HCC1937 (**C**) and HCC38 (**D**) treated with DMSO or AKT VIII. *P < 0.05. Data are mean ± SEM. *n* = 3 per group. **E** The scatter plot for the correlation between pAKT_308, protein expression (RPPA) and *WDHD1*, mRNA expression (IlluminaHiSeq) in the TCGA breast invasive carcinoma (Provisional) data (Pearson’s correlation (*r*) = 0.3321; *P* = 0.0296; *n* = 43).

Given our findings that decreased PTEN levels are responsible for the high AKT activity in TNBC, we then determined if AKT is involved in the regulation of WDHD1 expression in TNBC cells. An AKT inhibitor (AKT VIII) was used to treat PTEN null type TNBC cell lines MDA-MB-468 (Fig. 4A), HCC1395 (Fig. 4B), HCC1937 (Fig. 4C) and HCC38 (Fig. 4D). AKT activity, monitored by the levels of phosphorylated AKT (pAKT Thr308 and Ser473), was inhibited following the treatment with AKT VIII in all PTEN null type TNBC cell lines (Fig. 4A−D). Subsequently, WDHD1 levels were significantly reduced upon AKT inhibition in these cells (Fig. 4A−D; *P* < 0.05). The impact of PTEN-AKT signalling on WDHD1 expression was further confirmed by the TCGA analysis. To reflect the functional consequence of PTEN status, we decided to check p-AKT_308 levels and the correlation with *WDHD1* expression in TCGA. We demonstrated that there was a significant positive correlation between *WDHD1*, mRNA expression and pAKT_308 levels in the TCGA dataset (Fig. 4E; *r* = 0.3321, *P* = 0.0296).

Taken together, our results demonstrate that WDHD1 expression is affected by PTEN status in TNBC cells and this is mainly achieved by AKT signalling.

### WDHD1 is required for the survival of PTEN null TNBC cells cultured in 2D or 3D

**Fig. 5:**
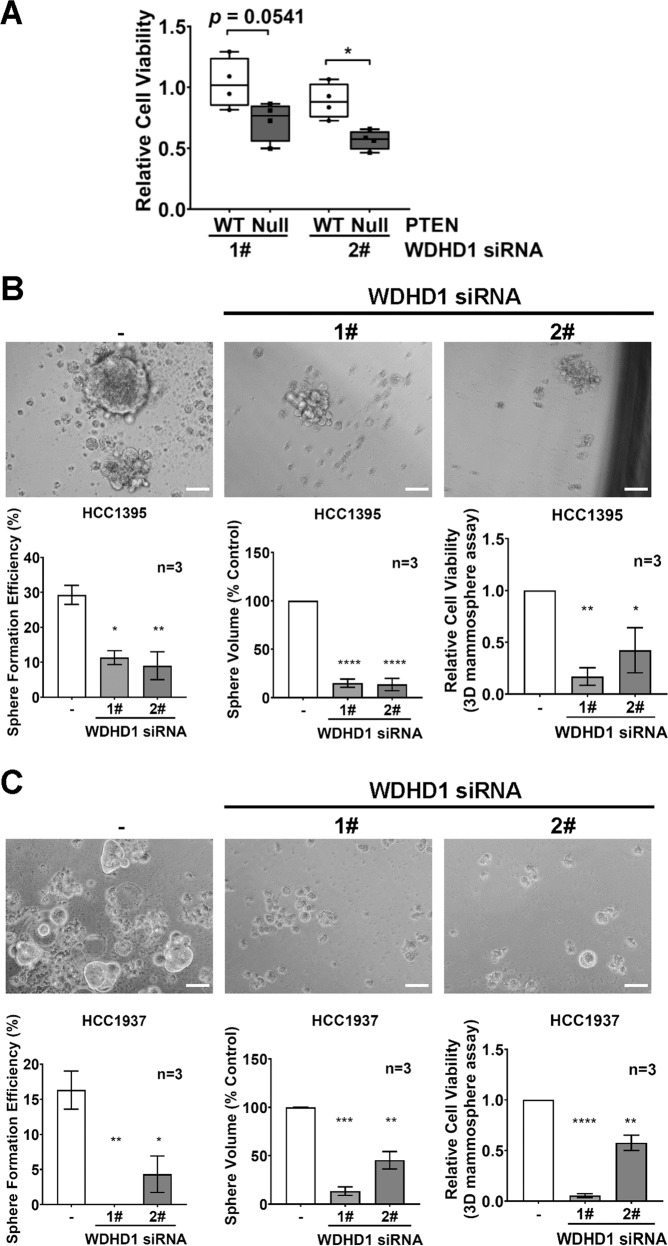
WDHD1 is required for the survival of PTEN null TNBC cells cultured in 2D or 3D. **A** Graph showing relative cell viability in PTEN WT or PTEN null TNBC cell lines transfected with control or *WDHD1* siRNAs in 2D cultures. Cell-Titer Glo® assay was performed to measure cell viability. Representative phase contrast microscopy images of PTEN null type TNBC cell line HCC1395 (**B**) or HCC1937 (**C**) with indicated transfections cultured in 3D. Scale bar: 50 µm. Graphs showing sphere formation efficiency, sphere volume and cell viability (Cell-Titer Glo® assay) in HCC1395 (**B**) or HCC1937 (**C**) with indicated transfections cultured in 3D. Data are mean ± SEM. *n* = 3 samples per group. *P < 0.05. **P < 0.01. ***P < 0.001. ****P < 0.0001.

The initial whole-genome siRNA screen suggested that *WDHD1* depletion selectively inhibits cell viability in PTEN-negative vs. -positive TNBC cells. To validate this observation, *WDHD1* expression was down-regulated by two individual siRNA oligos in the aforementioned panel of TNBC cell lines and cell viability was measured by Cell-Titer Glo^®^ assays (Supplementary Fig. S4). Knockdown of *WDHD1* in PTEN WT TNBC cell lines (HCC1806, BT20, MDA-MB-157 and MDA-MB-231) showed mild, but not significant, effects on cell viability (Supplementary Fig. S4a−d; *P* > 0.05). On the other hand, *WDHD1* knockdown in PTEN null type TNBC cell lines (MDA-MB-468, HCC1395 and HCC1937) showed a significant decrease in cell viability (Supplementary Fig. S4e−g). Although there was a reduction in cell viability with *WDHD1* knockdown in HCC38 cells, no significant difference was observed (Supplementary Fig. S4h). In general, consistent with the whole-genome siRNA screen, depletion of *WDHD1* selectively inhibited cell viability in PTEN null vs. WT TNBC cells with two individual siRNA oligos against *WDHD1*, although statistical significance for oligo 1# was not reached (*P* = 0.054) (Fig. 5A).

It is known that 3D cell cultures represent their in vivo counterparts better than 2D monolayer cell culture models^23,24^. To further validate the effects of *WDHD1* knockdown in TNBC cells, 3D mammosphere assays with PTEN WT (BT20 and MDA-MB-231) and null type (HCC1395 and HCC1937) TNBC cell lines were performed. Images of spheres were analysed for sphere formation efficiency and sphere volume, and cell viability was determined using Cell-Titer Glo^®^ assays. *WDHD1* depletion in PTEN WT TNBC cell lines (BT20 and MDA-MB-231) showed minimal effects on sphere formation efficiency, sphere volume and cell viability (Supplementary Fig. S5). In contrast, a significant decrease in sphere formation efficiency, sphere volume and cell viability with two individual siRNA oligos against *WDHD1* was observed in HCC1395 (Fig. 5B; *P* < 0.05) and HCC1937 (Fig. 5C; *P* < 0.05), both of which are PTEN null type TNBC cell lines.

These experiments showed that WDHD1 is preferentially required by PTEN-inactive TNBC cells for survival, but not for those harbouring WT PTEN.

### Essential roles of WDHD1 in cell cycle in PTEN null TNBC cell lines

In order to understand the functions of WDHD1, 92 TNBC samples from the TCGA were identified (Fig. 1A). The top 10% and bottom 10% of samples were separated into two groups: high and low *WDHD1* expressing samples, respectively, and those genes with *P* values < 0.05 were considered as differentially expressed genes (DEGs). A heatmap of 3796 DEGs in the high vs. low *WDHD1* groups (*P* < 0.05) was shown in Supplementary Fig. S6a. To investigate whether the significantly up-regulated 2069 genes in the high *WDHD1* group were enriched in certain cellular functions, ToppGene, (https://toppgene.cchmc.org/), was used. We found that the regulation of cell cycle was enriched in the high *WDHD1* TNBC samples (Supplementary Fig. S6b).

To validate these findings, *WDHD1* expression was depleted by two individual siRNA oligos in TNBC cell lines, followed by cell cycle analysis based on flow cytometry (Supplementary Fig. S7). Interestingly, depletion of *WDHD1* with two individual siRNA oligos significantly reduced the percentage of cells in S phase in PTEN null TNBC cells, including MDA-MB-468 (Supplementary Fig. S7a) and HCC1395 (Supplementary Fig. S7b). However, no effects on cell cycle were observed in PTEN WT TNBC cell lines, including BT20 (Supplementary Fig. S7c) and MDA-MB-231 (Supplementary Fig. S7d).

These results suggested an important role of WDHD1 in cell cycle regulation in PTEN null TNBC cell lines, consistent with the findings in cell viability assays.

### Essential roles of WDHD1 in protein translation in PTEN null TNBC cells

**Fig. 6:**
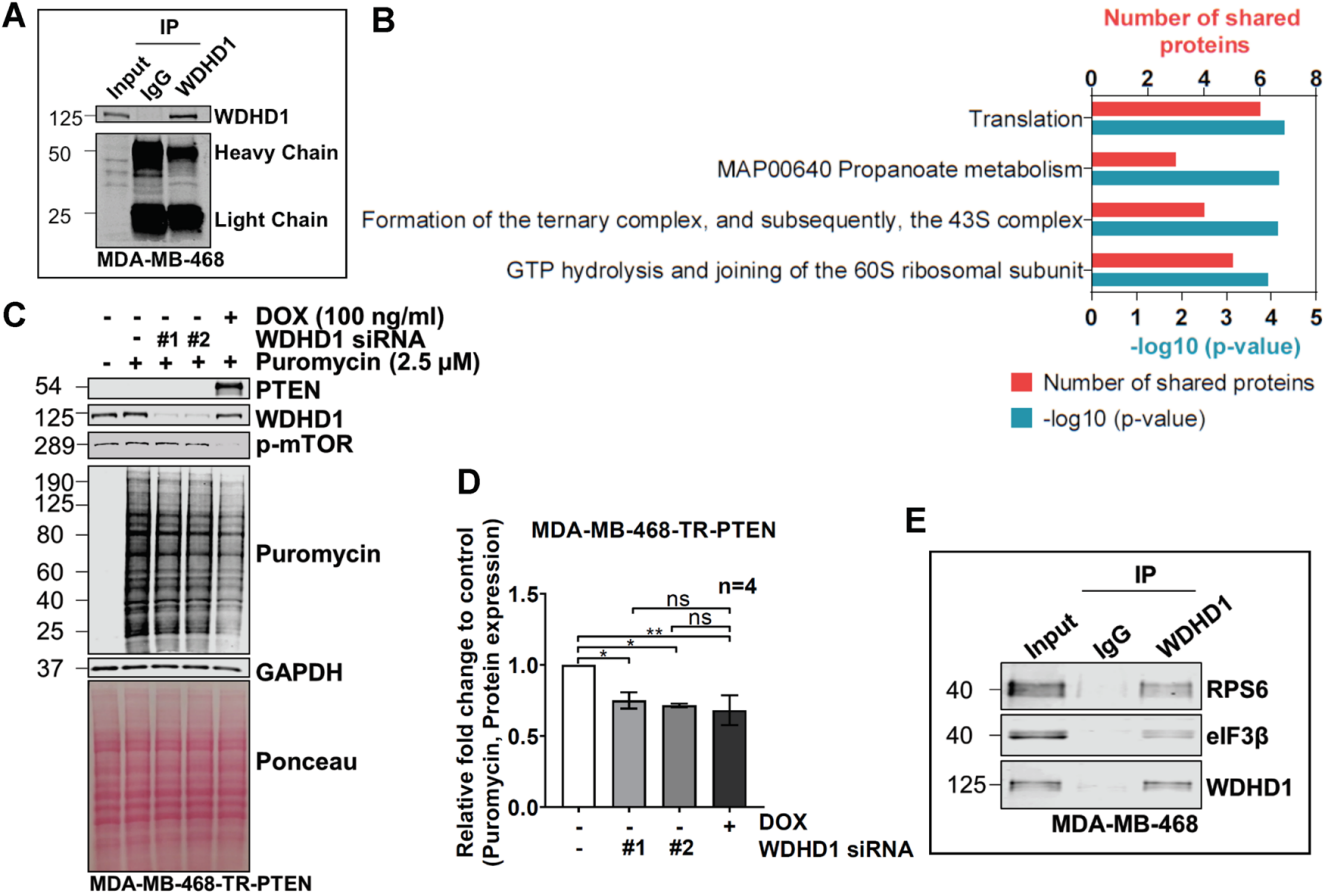
Essential roles of WDHD1 in protein translation in PTEN null TNBC cells. **A** Total cell lysates from MDA-MB-468 cell were immunoprecipitated with an anti-WDHD1 antibody or control IgG. WDHD1, IgG heavy and light chains are indicated. **B** Functional enrichment (ToppGene) of WDHD1 binding partners identified from an immunoprecipitation-mass spectrometry (IP-MS) experiment is visualised on a bar chart, showing number of shared proteins and −Log10 (*P* value). *P* values < 0.0001 are shown. **C** Puromycin labelling to measure protein synthesis in MDA-MB-468-TR-PTEN cells with indicated treatments. Equal amounts of total protein extracts were analysed by western blotting showing levels of PTEN, WDHD1, phospho-mTOR (p-mTOR) and puromycin labelling. GAPDH was used as a loading control. Ponceau S staining showing total protein levels. **D** Graph showing relative puromycin labelling intensity in MDA-MB-468-TR-PTEN cells with indicated treatments. Data are mean ± SEM. *n* = 4 samples per group. *P < 0.05. **P < 0.01. n.s. not significant, *P* > 0.05. **E** Total cell lysates from MDA-MB-468 cell were immunoprecipitated with an anti-WDHD1 antibody or control IgG. RPS6, eIF3β and WDHD1 are indicated.

By performing immunoprecipitation-mass spectrometry (IP-MS) analysis, we identified 64 proteins as WDHD1 binding partners in PTEN null MDA-MB-468 cells. Endogenous WDHD1 was immunoprecipitated along with control IgG as a negative control in MDA-MB-468 cells (Fig. 6A) followed by mass spectrometry analysis. Functional enrichment (ToppGene) of WDHD1 binding partners showed a total of 17 functions identified (Supplementary Table S3). The top four functions are shown in Fig. 6B, with protein translation as the top one (Fig. 6B), which suggests a role of WDHD1 in protein translation in PTEN null TNBC cells.

To verify these findings, *WDHD1* expression was depleted by two individual siRNA oligos in MDA-MB-468-TR-PTEN cells followed by puromycin incorporation assay to measure protein synthesis. Puromycin is commonly used to study translation^25,26^. Puromycin incorporation stops translation elongation and subsequently induces the release of puromycylated peptides from the ribosome^27^. Unlike radiolabelled amino acids and non-canonical amino acid analogues, puromycin incorporation is not significantly impacted by the endogenous methionine concentration nor the methionine content of proteins^26^. Puromycin thus incorporates relatively equally into all nascent polypeptides, making it a reliable tool for measuring global protein synthesis.

In this study, we utilised the puromycin incorporation assay, in which cells were treated with 2.5 µM puromycin for 5 min before sample collection. We were able to show a 25−30% reduction in global protein translation upon PTEN re-introduction or *WDHD1* depletion (Fig. 6C, D; *P* < 0.05). As a positive control, PTEN expression was induced in MDA-MB-468-TR-PTEN cells by addition of DOX, since it is known that PTEN inhibits protein translation through negative regulation of mammalian target of rapamycin (mTOR) (Fig. 6C)^28^.

As shown in Fig. 6C and D, depletion of *WDHD1* with two individual siRNA oligos significantly inhibited global protein translation in MDA-MB-468 cells, reflected by the reductions in the puromycin labelling intensity (Fig. 6D; *P* < 0.05). The inhibitory effect of *WDHD1* depletion on protein translation was similar to those achieved by re-introducing PTEN in MDA-MB-468 cells (Fig. 6C, D), indicating an important role of WDHD1 in protein translation in PTEN null TNBC cells. Interestingly, the phosphorylation level of mTOR was not affected by WDHD1 status (Fig. 6C), indicating that the impact of WDHD1 on protein translation is independent of mTOR. We further validated several interactions of WDHD1 with the potential binding partners (including RPS6 and eIF3β) identified via the IP-MS analysis (Fig. 6E), highlighting the interactions between WDHD1 and the components of translational machinery.

### WDHD1 levels are increased in TNBC compared to normal breast tissues, and associate with tumour size and proliferation

**Fig. 7:**
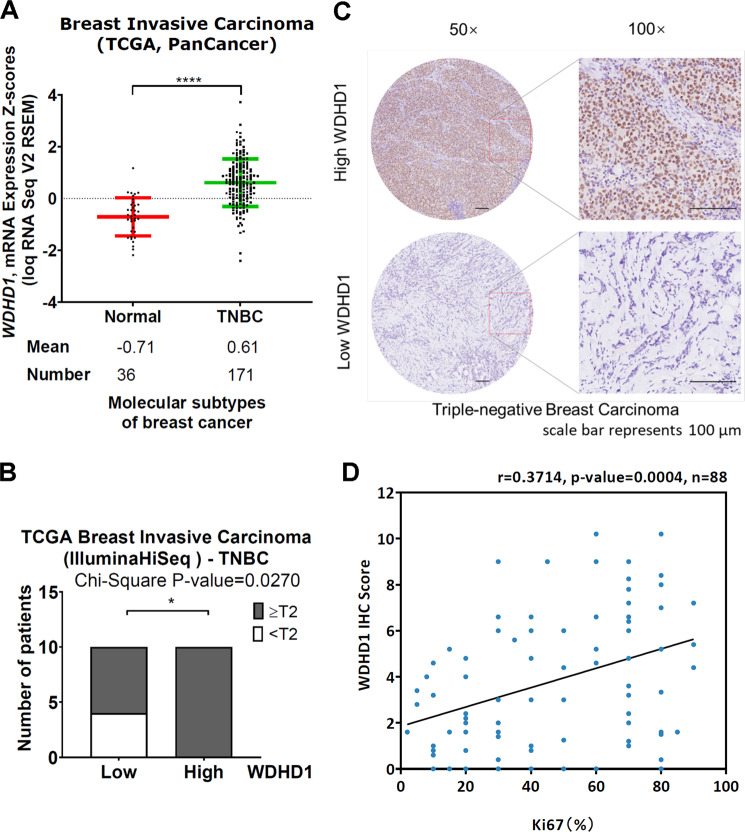
WDHD1 levels are increased in TNBC compared to normal breast tissues, and associates with tumour size and proliferation. **A** Graph showing *WDHD1*, mRNA levels (*Z* scores) in the normal breast (*n* = 36) and TNBC (*n* = 171) samples obtained from the TCGA data. Data are mean ±SD. ****P < 0.0001. **B** Graph showing the number of TNBC patients (TCGA) with T2 and above or < T2 in the low or high *WDHD1* group. *P < 0.05. **C** Representative WDHD1 staining pattern (high or low WDHD1) in TNBC tissue microarray cores. Scale bar: 100 μm. **D** The scatter plot for the correlation between WDHD1 scores and percentage of Ki67-positive cells in TNBC samples (Pearson’s correlation *r* = 0.3714; *P* = 0.0004; *n* = 88).

The clinical importance of WDHD1 in TNBC was evaluated in samples from TNBC patients. From TCGA analysis, *WDHD1* mRNA levels were significantly higher in TNBC than the normal breast samples (Fig. 7A; *P* < 0.0001). In addition, the number of patients with T2 and above in the high *WDHD1* group was significantly larger than the low *WDHD1* group (Fig. 7B; *P* = 0.027).

The association between WDHD1 and clinic-pathological features in TNBC patients was further investigated by immunohistochemistry (IHC) staining of WDHD1 in a TNBC tissue microarray. We found that tumour grade (*P* = 0.03) and tumour size (*P* = 0.016) were significantly correlated with WDHD1 expression (Table 1). Representative images of high and low expression of WDHD1 in TNBC are shown in Fig. 7C. Moreover, a positive correlation between WDHD1 expression levels (reflected by its IHC scores) and Ki67 percentage (a proliferation marker) was observed in TNBC (Fig. 7D; Pearson’s correlation *r* = 0.3714; *P* = 0.0004), suggesting a role of WDHD1 in regulating cell viability, in consistence with the above in vitro findings.

**Table 1:** The relationship between patients’ clinical–pathological characteristics and WDHD1 expression in TNBC.

Characteristics	*N*	WDHD1		*P* value
		Low expression	High expression	
Age	90			
≤ 50	46	26	20	0.686
>50	44	23	21	
Location	90			
Left breast	46	25	21	0.985
Right breast	44	24	20	
Grade	90			
I-II	36	25	11	***0.030***
III	54	24	30	
Size	86			
≤2 cm	37	26	11	***0.016***
2 cm	49	21	28	
Positive LN	35			
≤2	21	9	12	1.000
>2	14	6	8	

*P* values were calculated by *χ*^2^ test or Fisher’s exact test, if appropriate. Numbers in bold-italics mean *P* values less than 0.05 and are statistically significant.

*LN* lymph.

## Discussion

As TNBC is difficult to be targeted and is molecularly heterogeneous, further stratification is needed. TNBC has been subdivided into six distinct subtypes: basal-like 1 (BL1), basal-like 2 (BL2), immunomodulatory (IM), mesenchymal (M), mesenchymal stem-like (MSL) and luminal androgen receptor (LAR)^9^. Another study re-classified TNBC into five stable subtypes: BL1, IM, M, MSL and LAR^29^. PTEN inactivation was observed in the BL1 subtype^29^, which was further confirmed in a recent in silico analysis, showing exceedingly poor clinical outcome^30^.

Loss-of-function mutations in TSGs, such as *PTEN*, are major genetic alterations leading to more challenges to identify targeted drugs since it is difficult to restore their functions^31^. Therefore, studies have been focused to target downstream signalling pathways that are altered by inactivation of TSGs^18,31^. Targeting synthetic lethality provides an alternative approach^32^. As the second most mutated gene following p53 in various cancer types^33^, various studies have been performed to identify *PTEN* synthetic lethal interactions in a variety of cancer types. These include mitochondrial complex I inhibitors^34^ and chromatin helicase DNA-binding factor *CHD1* in PTEN-inactive prostate cancer cells^35^, polynucleotide kinase/phosphatase (*PNKP*) in PTEN-deficient lung and colon cancer cells, and NUAK family kinase 1 (*NUAK1*) in PTEN-deficient breast cancer cells^36^. In this study, using TCGA analysis coupled with a whole-genome siRNA screen in isogenic PTEN-negative and -positive TNBC cells, we identified *WDHD1* as a synthetic essential gene in PTEN-inactive TNBC cells.

*WDHD1*, an orthologue of *Ctf4* in budding yeast^37^ and *Mcl1* in fission yeast^38^, is a DNA-binding protein^39^ that is known to play important roles in DNA replication and cell cycle^37,40–46^. We also observed an important role of WDHD1 in cell cycle, especially in PTEN-inactive TNBC cells. The selective killing of *WDHD1* depletion in PTEN-inactive TNBC cells was further validated in both 2D and 3D cultures. In addition, using IP-MS analysis followed by bioinformatics, we identified a potential, yet unknown function of WDHD1 in protein translation in PTEN null TNBC cells, which was further validated with puromycin incorporation assay to measure global protein synthesis. Depletion of *WDHD1* significantly inhibits global protein translation in PTEN null TNBC cells, which is independent of mTOR inhibition and potentially via directly interacting with the translational machinery. The impact of WDHD1 depletion on global protein translation is similar to the effect achieved by re-introducing PTEN. PTEN inactivation in TNBC leads to a high activity of mTOR^47^, which is linked to a high rate of protein synthesis, creating an “Achilles heel” of TNBC. Indeed, several clinical trials on Everolimus (a mTOR inhibitor) in TNBC are ongoing (clinicaltrials.gov), some of which showed positive results^48,49^. However, a common pattern seen in trial data is of a modest response to rapalog (rapamycin and its analogues) monotherapy, which does not lead to a significant improvement in patient outcomes. One of the likely reasons is that it is caused by reactivation of signalling pathways that drive the high rate of protein synthesis required by tumour growth. Inhibition of WDHD1 in a PTEN-inactive background reduces protein translation, suggesting that such a “synthetic sickness” approach may be applicable to PTEN-deficient tumours when rapalog resistance happens.

In addition, a potential role of WDHD1 in regulating the stemness of PTEN-inactive TNBC cells was investigated using a mammosphere formation assay, which is one of the assays used to determine cell stemness^50^. Given the impact of WDHD1 on cell cycle and protein translation, both of which play important roles in regulating cell stemness^51^, we presume that WDHD1 may control stemness in PTEN-inactive TNBC cells via its ability to regulate cell cycle and protein translation; however, this remains to be elucidated. We found WDHD1 expression is significantly higher in PTEN-inactive TNBC cells than in PTEN-active ones. A previous report from Sato et al.^44^and colleagues suggested that AKT kinase seems to phosphorylate and stabilise the WDHD1 protein in cancer cells. In addition to the reported effects of AKT on WDHD1 protein stability, we found the mRNA levels of *WDHD1* are also regulated by the PTEN-AKT pathway. Together, these data suggest that WDHD1 expression is affected by PTEN-AKT signalling in TNBC cells at both mRNA and protein levels.

The clinical importance of WDHD1 in TNBC was evaluated in samples obtained from TNBC patients, showing that its levels are increased in TNBC compared to normal breast tissues, and associates with tumour size, stage and proliferation, using Ki67 as a proliferation marker^52^. Moreover, recent reports demonstrated that overexpression of WDHD1 leads to cisplatin resistance in lung adenocarcinoma^53^ and metastasis in cholangiocarcinoma^54^. Further studies are required to confirm these findings in TNBC. The data presented here suggest that inhibitors that can disrupt the interactions between WDHD1 and the protein synthesis machinery could target some of the most intractable tumour types, such as TNBC with PTEN-deficiency. The relatively mild effects of *WDHD1* depletion in PTEN-positive cells suggests that on-target inhibition of this factor may also be relatively free from unwanted side effects.

## Materials and methods

### Cell culture, transfections and reagents

Human breast cancer cell lines (HCC1806, BT20, MDA-MB-157, MDA-MB-231, MDA-MB-468, HCC1395, HCC1937 and HCC38) were obtained as NCI-ICBP45 kit procured through American Type Culture Collection (ATCC) (ATCC Breast Cancer Cell Panel, Manassas, VA, USA). Cell lines were authenticated by ATCC using short tandem repeat DNA profiling, and each cell culture was examined by light microscopy and compared with images published by ATCC and the Integrative Cancer Biology Program (ICBP; http://icbp.lbl.gov/breastcancer/celllines.php) to verify identity^55^. HCC1806, HCC1395, HCC1937 and HCC38 cells were maintained in Roswell Park Memorial Institute (RPMI) 1640 medium, (Gibco^®^ by Life Technology) with 10% fetal bovine serum (FBS) and 1% (v/v) penicillin/streptomycin, (Gibco^®^ by Life Technology). BT20, MDA-MB-157, MDA-MB-231, MDA-MB-468 and MDA-MB-468-TR-PTEN cell lines were maintained in Dulbecco’s modified Eagle’s medium (DMEM) (Gibco^®^ by Life Technology) with 10% FBS and 1% (v/v) penicillin/streptomycin. All cells were kept at 37 °C and 5% CO_2_. No mycoplasma contamination was detected in the cell lines used. AKT VIII and puromycin were from Sigma-Aldrich.

For PTEN-inducible cells, MDA-MB-468 cells were stably transfected with a tetracycline-inducible PTEN vector and named MDA-MB-468-TR-PTEN, in which addition of Doxycycline (DOX) acutely induces PTEN expression. MDA-MB-468 cells were also stably transfected with a tetracycline-inducible parent vector and used as vector-only controls (MDA-MB-468-TR-EV). To fluorescently label MDA-MB-468-TR-PTEN and MDA-MB-468-TR-EV cells, pCherryFP-N1 and p-EGFP-N1 were stably transfected into them, respectively. Single clones were picked and sorted by fluorescence-activated cell sorting (FACS), and named as MDA-MB-468-TR-PTEN/CherryFP or MDA-MB-468-TR-EV/GFP.

Short-interfering RNA (siRNA) oligos against WDHD1 *(D-019780-02* and *D-019780-03)* was purchased from Dharmacon. Sequences are available from Dharmacon, or upon request. siGENOME RISC-Free siRNA (Dharmacon) was used as a negative control. Cells were transfected with the indicated siRNA oligos at a final concentration of 35 nM using Dharmafect 2 reagent (Dharmacon).

### The Cancer Genome Atlas (TCGA) data analysis

Expression of genes/proteins of interest, obtained from the cBioPortal for Cancer Genomics (https://www.cbioportal.org/) and UCSC Cancer Genome Browser (https://genome-cancer.ucsc.edu/), were analysed in each breast cancer molecular subtype along with normal breast samples (details provided in Supplementary Methods).

### A whole-genome siRNA screen and data analysis

The human siGENOME siRNA library—Genome (G-005005) was obtained from Dharmacon. siRNA transfection experiments were performed in 96-well format in antibiotic-free medium, using a reverse transfection employing 25 nM siRNA and 0.15 μl Dharmafect 2 (Dharmacon) per well together with a starting cell density optimised to produce an 80% confluent monolayer in mock-treated cells at the conclusion of the experiment. DOX-treated MDA-MB-468-TR-PTEN/CherryFP (PTEN+) or MDA-MB-468-TR-EV/GFP (PTEN−) cells were mixed and transfected at a 1:1 ratio in 96-well plates. Cells were fixed with 4% paraformaldehyde at 96 h post transfection. Fluorescence was read on an EnVision 2102 Plate-reader (Perkin-Elmer).

Triplicate data points from CherryFP channel (PTEN+) and GFP channel (PTEN−) screens underwent plate and position normalisation and *Z* score calculation using cellHTS software^56,57^. Differential *Z* scores (Δ*Z* score) between the two channels were subsequently used to create a gene hit list. Reproducibility of the replicates was analysed by performing Pearson correlation analysis in GraphPad Prism 8. *P* value < 0.05 was considered significant (details provided in Supplementary Methods).

### Cell viability assay

siRNA transfected cells were plated into 96-well plate with a density of 8000 cells/well. CellTiter-Glo^®^ Luminescent cell viability assay (Promega) was performed 96 h post transfection according to the manufacturer’s protocol using GloMax^®^ Discover Microplate Reader (Promega).

### Mammosphere assay and quantifications

siRNA transfections were performed in 2D cultures. Ninety-six hours post transfections, cells were cultured in 96-well ultralow attachment plate in 100 µl at plating densities between 3000 and 7000 cells/well. Cells were cultured in 1:1 DMEM:F12, (Gibco^®^ by Life Technology) media plus 1% P/S, 2% B27 (Gibco^®^ by Life Technology), 20 ng/ml epidermal growth factor (EGF), (PEPROTECH) and 20 ng/ml basic-fibroblast growth factor (bFGF) (PEPROTECH) at 37 °C and 5% CO_2_ for 14 days. After the incubation period, the images were taken using with ×40 magnification.

The mammospheres that were equal to or greater than 50 μm in diameter were counted to calculate the mammosphere formation efficiency (MFE%) with the following equation: (# of mammospheres per well)/(# of cells seeded per well) × 100. Additionally, the volumes of the mammospheres were also calculated using the formula of Volume = (4/3)*πr*^3^. ImageJ (version1.42q) was used to determine the MFE and volume of sphere.

CellTiter-Glo^®^ cell viability assay was performed with addition of 100 µl of CellTiter-Glo^®^ reagent into each well and incubated at room temperature for 1 h, followed by measuring using GloMax^®^ Discover Microplate Reader (Promega).

### Western blot analysis

Western blot analysis was performed with lysates from cells lysed with urea buffer (8 M urea, 1 M thiourea, 0.5% CHAPS, 50 mM 1,4-Dithiothreitol (DTT) and 24 mM spermine). The bound proteins were separated on sodium dodecyl sulphate (SDS) polyacrylamide gels and subjected to immunoblotting with the indicated antibodies. For immunoprecipitations, the cells were lysed for 30 min at 4 °C in pNAS buffer (50 mm Tris/HCl (pH 7.5), 120 mm NaCl, 1 mm ethylenediaminetetraacetic acid (EDTA) and 0.1% Nonidet P-40), with protease inhibitors. Anti-WDHD1 (Sigma-Aldrich) or control antibodies and Protein G magnetic beads (Thermo Fisher Scientific) were added to the lysate for 16 h at 4 °C. Immunoprecipitates were washed four times with cold phosphate buffered saline (PBS) followed by the addition of SDS sample buffer. The bound proteins were separated on SDS polyacrylamide gels and subjected to immunoblotting with the indicated antibodies. Primary antibodies were from Cell Signalling Technology (PTEN (D4.3) XP^®^, 1:1000, 9188; phospho-AKT (Thr308) (244F9), 1:1000, 4056; phospho-AKT (Ser473), 1:1000, 9271; AKT, 1:1000, 9272; Phospho-ERK, 1:1000, 9101; ERK, 1:1000, 9102; Phospho-mTOR (Ser2448), 1:1000, 2971; β-tubulin (D3U1W), 1:1000, 86298), Sigma-Aldrich (WDHD1, 1:500, HPA001122; Puromycin, 1:2000, MABE343), PROTEINTECH (GAPDH, 1:10,000, 10494-1-AP), Santa Cruz Biotechnology (RPS6 (C-8), 1:500, sc-74459; eIF3β (A7), 1:500, sc-374156). Signals were detected using an Odyssey imaging system (LI-COR) or an ECL detection system (GE Healthcare, Chicago, IL, USA), and evaluated by ImageJ (version1.42q) software (National Institutes of Health) (Berhesda, MD, USA).

### qRT-PCR

RNA extraction was performed by RNeasy^®^ Mini Kit (Qiagen) manufacturer’s protocol and Nanodrop Spectrophometer 2000c (Thermo Fisher Scientific) was used to quantify RNA concentration. QuantiNova™ SYBR Green RT-PCR kits (Qiagen) were used with *WDHD1* (QT00062244) and *ACTB* (β-actin, QT00095431) gene-specific primers (QuantiTect Primer Assays, Qiagen). Relative mRNA levels of target genes were normalised to *ACTB* (β-actin).

### Immunofluorescence microscopy

Cells were fixed in 4% PBS-paraformaldehyde for 15 min, incubated in 0.1% Triton-X-100 for 5 min on ice, then in 0.2% Fish Skin Gelatine in PBS for 1 h and stained for 1 h with an anti-WDHD1 (1:500, Sigma-Aldrich, HPA001122). Protein expression was detected using Alexa Fluor (1:400, Molecular Probes) for 20 min. 4'6-Diamidino-2-Pheylindole (DAPI) (Invitrogen) was used to stain nuclei (1:1000). Samples were observed using a confocal microscope system (Leica SP8). Acquired images were analysed using Fiji^58^.

### Immunohistochemical and H/E staining and scoring

Tissue microarray of TNBC patients with information of clinic-pathological parameters was purchased from Outdo Biotech (HBreD090Bc01; Shanghai, China). Tissue samples were pre-stained with Ki67. All procedures were approved by the Ethical Committee of Tongji Hospital, China. Informed consent was obtained from all subjects. For immunohistochemical staining, antigen retrieval, blocking of non-specific binding and incubation of primary antibodies at 4 °C overnight were conducted sequentially. The primary antibody of anti-WDHD1 (HPA001122, Sigma-Aldrich, 1:500) was used. After incubation with secondary goat anti-rabbit immunoglobulin conjugated to peroxidase-labelled dextran polymer (SV0002; Boster) at 37 °C for 1 h, visualisation, counterstaining with haematoxylin and mounting were performed. Semi-quantitative evaluations of protein expression were scored on the basis of the intensity and the percentage of WDHD1-positive tumour cells as previously described^59–62^.

### Flow cytometry

For cell cycle analysis, 48 h post transfection, cells were fixed with 70% ethanol and kept at 4 °C for up to 2 weeks. Cells were treated with 0.25% Triton-X-100, 200 µg/ml RNAse A and 50 µg/ml propidium iodide (PI), and analysed by FACS, Guava.

### Immunoprecipitation-mass spectrometry (IP-MS) analysis

For immunoprecipitations of endogenous WDHD1, the cells were lysed for 30 min at 4 °C in pNAS buffer (50 mm Tris/HCl (pH 7.5), 120 mm NaCl, 1 mm EDTA and 0.1% Nonidet P-40), with protease inhibitors. Anti-WDHD1 (Sigma-Aldrich) or control antibodies and Protein G Sepharose (GE Healthcare) were added to the lysate for 16 h at 4 °C. Immunoprecipitates were washed four times with cold PBS followed by mass spectrometry analysis (details provided in Supplementary Methods).

Two repeats of WDHD1 and two repeats of IgG control samples were combined in RStudio (version 3.4.4), and the proteins with *NA* values in more than two samples were removed. The average of peptide numbers for WDHD1 and IgG control samples was calculated and ratio of peptide numbers for each sample group was calculated. The proteins which had two times higher peptide number in WDHD1 compared to the control samples were chosen as threshold and used to perform pathway analysis in ToppGene website as described below.

### Bioinformatics

For pathway analysis, ToppGene Suite (https://toppgene.cchmc.org/) was used to detect functional enrichment of the mRNAs or proteins. The pathways were sorted from lowest *P* value and top 15 pathways were chosen for TCGA data. We then produced a histogram plot with the top 15 pathways in GraphPad Prism 8. The pathways for IP-MS data were sorted from lowest *P* value and the histogram was plotted with top four pathways in GraphPad Prism 8.

### Statistical analysis

Two tailed, unpaired Student’s *t* test for the TCGA data and two paired, paired Student’s *t* test for the whole-genome siRNA screening data were performed in RStudio (version 3.4.4). Codes are available upon request. Unless stated otherwise, comparison of two groups was statistically calculated by two paired, unpaired Student’s *t* test in GraphPad Prism 8 software. Ordinary one-way ANOVA was conducted to statistically compare more than two groups in GraphPad Prism 8 software. Correlation analysis was conducted by Pearson’s correlation in GraphPad Prism 8 software. *χ*^2^ test was used to analyse the association of PTEN and WDHD1 with clinical features of TNBC samples in the TCGA breast invasive carcinoma data in GraphPad Prism 8 software. *χ*^2^ test or Fisher’s exact test was used to evaluate the relationship of WDHD1 and clinic-pathological parameters of TNBC patient samples in IHC using SPSS (version 19.0). Data were shown as box and whisker plot with minimum and maximum individual values, mean ± SD or mean ± SEM, indicated in figure legend.

## Supplementary information

Supplementary Text

Supplementary Figure 1

Supplementary Figure 2

Supplementary Figure 3

Supplementary Figure 4

Supplementary Figure 5

Supplementary Figure 6

Supplementary Figure 7

Supplementary Table 1

Supplementary Table 2

Supplementary Table 3
