# Correction: WDHD1 is essential for the survival of PTEN-inactive triple-negative breast cancer

**DOI:** 10.1038/s41419-021-03530-0

**Published:** 2021-03-15

**Authors:** Ayse Ertay, Huiquan Liu, Dian Liu, Ping Peng, Charlotte Hill, Hua Xiong, David Hancock, Xianglin Yuan, Marcin R. Przewloka, Mark Coldwell, Michael Howell, Paul Skipp, Rob M. Ewing, Julian Downward, Yihua Wang

**Affiliations:** 1 Biological Sciences, Faculty of Environmental and Life Sciences, University of Southampton, Southampton, SO17 1BJ UK; 2 Department of Oncology, Tongji Hospital, Tongji Medical College, Huazhong University of Science and Technology, 430030 Wuhan, China; 3 Oncogene Biology, The Francis Crick Institute, London, NW1 1AT UK; 4 Institute for Life Sciences, University of Southampton, Southampton, SO17 1BJ UK; 5 High-Throughput Screening, The Francis Crick Institute, London, NW1 1AT UK; 6 Centre for Proteomic Research, Institute for Life Sciences, University of Southampton, Southampton, SO17 1BJ UK; 7 NIHR Southampton Biomedical Research Centre, University Hospital Southampton, Southampton, SO16 6YD UK

**Keywords:** Cell signalling, Breast cancer

Correction to: *Cell Death and Disease*

10.1038/s41419-020-03210-5 published online 21 November 2020

The original version of this article unfortunately contained a mistake. In the original publication, Supplementary Tables 1 and 2 were not the final version. This was a mistake made by the authors during the submission. This error does not affect any of the findings reported in the paper. The final version of Supplementary Tables 1 and 2 are now provided. The authors apologize for any confusion that this error may have caused. The original article has been corrected.

## Supplementary information

Supplementary Table 1

Supplementary Table 2

