# Supplementary material for: WDHD1 is essential for the survival of PTEN-inactive triple-negative breast cancer
Source: Cell Death Dis. 2020 Nov 21;11(11):1001. doi: 10.1038/s41419-020-03210-5 (PMC7680459; doi:10.1038/s41419-020-03210-5)
Supplement: Supplementary file 1 — Supplementary Text [file 41419_2020_3210_MOESM1_ESM.docx]

**WDHD1 is essential for the survival of PTEN-inactive triple negative breast cancer**

Ayse Ertay^1^, Huiquan Liu^2^, Dian Liu^2^, Ping Peng^2^, Charlotte Hill^1^, Hua Xiong^2^, David Hancock^3^, Xianglin Yuan^2^, Marcin R. Przewloka^1,4^, Mark Coldwell^1,4^, Michael Howell^5^, Paul Skipp^1,4,6^, Rob M. Ewing^1,4^, Julian Downward^3,*^ and Yihua Wang^1,4,7,*^

# ^1^Biological Sciences, Faculty of Environmental and Life Sciences, University of Southampton, Southampton SO17 1BJ, UK; ^2^Department of Oncology, Tongji Hospital, Tongji Medical College, Huazhong University of Science and Technology, Wuhan 430030, China; ^3^Oncogene Biology, The Francis Crick Institute, London NW1 1AT, UK; ^4^Institute for Life Sciences, University of Southampton, Southampton, SO17 1BJ, UK; ^5^High-Throughput Screening, The Francis Crick Institute, London NW1 1AT, UK; ^6^Centre for Proteomic Research, Institute for Life Sciences, University of Southampton, Southampton, SO17 1BJ, UK; ^7^NIHR Southampton Biomedical Research Centre, University Hospital Southampton SO16 6YD, UK.

# *Correspondence should be addressed to JD (e-mail: Julian.Downward@crick.ac.uk) or YW (e-mail: yihua.wang@soton.ac.uk).

# Keywords

WDHD1, triple negative breast cancer, PTEN, siRNA screen, TCGA

# Supplementary Materials

## The Cancer Genome Atlas (TCGA) data mining of PTEN

*PTEN*, mRNA expression Z-scores and PTEN mutation status of breast invasive carcinoma (TCGA, PanCancer) were obtained from the cBioPortal for Cancer Genomics (<https://www.cbioportal.org/>). *PTEN*, mRNA expression was analysed in each breast cancer molecular subtype along with normal breast samples in GraphPad Prism 8.

Clinical data of breast invasive carcinoma (TCGA, Provisonal) was extracted from the cBioPortal for Cancer Genomics. Molecular subtypes of breast cancer samples were separated based on ER, PR and HER2 status in the clinical data and TNBC samples were extracted.

The two different data sets TCGA_BRCA_RPPA-2015-02-24 for protein expression (RPPA) and TCGA_BRCA_exp_HiSeqV2-2015-02-24 for mRNA expression (IlluminaHiSeq), were extracted from the UCSC Cancer Genome Browser (<https://genome-cancer.ucsc.edu/>) for the analysis of TNBC samples. TNBC samples in clinical data from the cBioPortal for Cancer Genomics website were aligned with the samples in the TCGA data for both protein and mRNA expression that were extracted from the UCSC Cancer Genome Browser in RStudio (version 3.4.4). Codes are available upon request.

The correlation between PTEN, protein expression in the TCGA protein data (RPPA) and *PTEN*, mRNA expression in the TCGA mRNA expression data (IlluminaHiSeq) was analysed by Pearson’s correlation analysis in TNBC samples that were subcategorized from breast invasive carcinoma (TCGA, Provisional).

## TCGA breast invasive carcinoma (Protein, RPPA) analysis

Protein (RPPA) TCGA breast invasive carcinoma data from the UCSC Cancer Genome Browser (<https://genome-cancer.ucsc.edu/>) was obtained that included 410 breast invasive carcinoma samples and 142 proteins. Breast invasive carcinoma samples from the Cancer Genome Browser were aligned with TNBC samples, which were categorised in breast invasive carcinoma (TCGA, Provisional) data. There were 43 common TNBC samples between these two data sets. PTEN, protein expression across the samples was narrowly distributed, samples were grouped as high and low PTEN expressing TNBC samples according to approximately the top 40% and bottom 40% of samples, respectively. The clinical features, tumour size and tumour stage were analysed between the high and low PTEN expressing TNBC samples. The correlation between PTEN and AKT1_PT308 protein expressions (TCGA, RPPA) in TNBC samples that were subcategorized from breast invasive carcinoma (TCGA, Provisional) was analysed by Pearson’s correlation analysis.

## TCGA breast invasive carcinoma (mRNA, IlluminaHiSeq) analysis

mRNA (IlluminaHiSeq) TCGA breast invasive carcinoma data from the UCSC Cancer Genome Browser (<https://genome-cancer.ucsc.edu/>) was obtained that included 1,215 breast invasive carcinoma samples and 20,530 mRNAs. Breast invasive carcinoma samples from the Cancer Genome Browser were aligned with TNBC samples, which were categorised in breast invasive carcinoma (TCGA, Provisional) data. There were 92 common TNBC samples between these two data sets. *PTEN*, mRNA expression across the samples was widely distributed. The TNBC samples were grouped into high and low *PTEN* expression based on approximately the top 10% and bottom 10% of samples, respectively. Then, analysis was performed to find the significantly different mRNAs (*P* < 0.05) between the high and low PTEN groups in RStudio (version 3.4.4). Codes are available upon request.

## A whole genome siRNA high-throughput screening and data analysis

To optimise the concentration of doxycycline (DOX), MDA-MB-468-TR-PTEN and MDA-MB-468-TR-EV cell lines were treated with different concentrations of DOX to induce PTEN expression. MCF10A is a non-tumorigenic triple negative breast cell line. PTEN expression was induced with the treatment of DOX in MDA-MB-468-TR-PTEN cell line compared to the DOX untreated cell line (control). No PTEN induction was observed with the treatment of DOX in MDA-MB-468-TR-EV cell line. Similar levels of PTEN induction were observed with different concentrations of DOX, comparable to the endogenous PTEN expression in MCF10A cells (Fig. S2a). In the following studies, we used 100 ng/ml DOX to induce PTEN in MDA-MB-468-TR-PTEN cells. As expected, PTEN induction led to reduced levels of phospho-AKT (p-AKT), but not phospho-ERK (p-ERK) (Fig. S2a).

To fluorescently label MDA-MB-468-TR-PTEN and MDA-MB-468-TR-EV cells, pCherryFP-N1 and p-EGFP-N1 were stably transfected into them, respectively. Single clones were picked and sorted by fluorescence-activated cell sorting (FACS), and named as MDA-MB-468-TR-PTEN/CherryFP or MDA-MB-468-TR-EV/GFP (Fig. S2b).

The human siGENOME siRNA library - Genome (G-005005) was obtained from Dharmacon. siRNA transfection experiments were performed in 96-well format in antibiotic-free medium, using a reverse transfection employing 25 nM siRNA and 0.15 μl Dharmafect 2 (Dharmacon) per well together with a starting cell density optimized to produce an 80% confluent monolayer in mock-treated cells at the conclusion of the experiment. DOX-treated MDA-MB-468-TR-PTEN/CherryFP (PTEN+) or MDA-MB-468-TR-EV/GFP (PTEN-) cells were mixed and transfected at a 1:1 ratio in 96-well plates. Cells were fixed with 4% paraformaldehyde at 96 h post transfection. CherryFP or GFP fluorescence was read on an EnVision 2102 Plate-reader (Perkin-Elmer) to evaluate cell numbers in PTEN+ or PTEN- cells, respectively (Fig. S2c). Overall, four patterns were observed, including “No effects”, “Non-selective cytotoxic”, “Cytotoxic hits for PTEN+” and “Cytotoxic hits for PTEN-” (Fig. S2c).

The whole genome siRNA screen data contained siRNAs targeting 21,121 genes in two cell lines expressing GFP fluorescence (PTEN-) or red fluorescence (PTEN+), respectively. Each group contained three biological repeats, showing reproducible results (Pearson’s correlation (r) = 0.8 and *P* < 0.001) (Fig. S3a). We identified 4,647 genes that have differential effects between PTEN- and PTEN+ expressing cells (*P* < 0.05), which were shown in a heat-map (Fig. S3b).

## Identification of top hit genes

The statistically different mRNAs in the TCGA (IlluminaHiseq) data set that were highly expressed in the low PTEN TNBC group were merged with statistically different genes in the whole genome siRNA screening data set, which showed a decrease in cell viability in PTEN- TNBC cell line group by using RStudio (version 3.4.4) to identify the top hit candidate gene(s).

## TCGA data mining with identified top hit gene, WDHD1

*WDHD1*, mRNA expression Z-scores of breast invasive carcinoma (TCGA, PanCancer) were obtained from the cBioPortal for Cancer Genomics. *WDHD1*, mRNA expression was analysed between the normal breast and TNBC samples in GraphPad Prism 8.

*WDHD1*, mRNA expression of subcategorized-TNBC samples using clinical data (TCGA, Provisional) was extracted from the TCGA breast invasive carcinoma (IlluminaHiSeq) data set. Approximately the top 10% and bottom 10% of TNBC samples were chosen for the high and low *WDHD1*, mRNA expression in IlluminaHiSeq data. Then, significantly different mRNAs were identified between the high *vs.* low *WDHD1* groups in RStudio (version 3.4.4), *P* < 0.05. Tumour size of TNBC samples from the clinical data (TCGA, Provisional) was extracted and analysed between the low and high *WDHD1*, mRNA expression.

## Sample preparations for mass spectrometry

Protein G Sepharose beads (GE Healthcare) were re-suspended in 100 µL of 100 mM ammonium bicarbonate containing 0.25% Rapigest (Waters Corporation), heated at 70°C for 60 min, centrifuged at 13,000 x g for 5 min and the supernatant was collected. Proteins extracts were reduced with 0.5 µg DTT for 1 h and then alkylated with 2.5 µg IAA for 45 min in the dark, and digested with 0.5 µg sequencing grade modified trypsin (1/50 (w/w)) overnight at 37°C. Samples were acidified with 1% trifluoroacetic acid (v/v), centrifuged at 13,000 x g for 5 min and the supernatant collected. Supernatants were lyophilized and re-suspended in 20 µL of buffer A (0.1% formic acid in water (v/v)) prior to mass spectrometry.

## Mass spectrometry and database search

18 µL of peptide extracts in buffer A were separated on an Ultimate 3000 RSLC nano system, (Thermo Scientific), using a PepMap C18 EASY-Spray LC column, 2 μm particle size, 75 μm x 75 cm column, (Thermo Scientific), over a 140 min (single run) linear gradient of 3–25% buffer B (0.1% formic acid in acetonitrile (v/v)) in buffer A (0.1% formic acid in water (v/v)) at a flow rate of 300 nL/min. Peptides were introduced using an EASY‐Spray source at 2000 V to a Fusion Tribrid Orbitrap mass spectrometer, (Thermo Scientific). The ion transfer tube temperature was set to 275°C. Full MS spectra were recorded from 300 to 1500 m/z in the Orbitrap at 120,000 resolution with an automatic was performed using TopSpeed mode at a cycle time of 3 s. Peptide ions were isolated using an isolation width of 1.6 amu and trapped at a maximal injection time of 120 ms with an AGC target of 300,000. Higher‐energy collisional dissociation (HCD) fragmentation was induced at an energy setting of 28 for peptides with a charge state of 2–4. Fragments were analysed in the orbitrap at 30,000 resolution.

Analysis of raw data was performed using Proteome Discoverer software (Thermo Scientific), and the data processed to generate reduced charge state and deisotoped precursor and associated product ion peak lists. These peak lists were searched against the Human protein database. A maximum of one missed cleavage was allowed for tryptic digestion and the variable modification was set to contain oxidation of methionine and N-terminal protein acetylation. Carboxyamidomethylation of cysteine was set as a fixed modification. The false discovery rate (FDR) was estimated with randomized decoy database searches and were filtered to 1% FDR.

**Supplementary Figure Legends**

**Figure S1. TCGA analysis confirms PTEN expression is decreased in TNBC and correlates with clinical stages.**

**a** Graph showing mRNA levels (Z-scores) of *PTEN* in the TCGA samples from normal breast (*n* = 36), luminal A (*n* = 499), luminal B (*n* = 197), HER2+ (*n* = 78) and TNBC (*n* = 171). Data are mean ± SD. *****P* < 0.0001. **b** The scatter plot for the correlation of TNBC samples between PTEN, protein expression (RPPA) and *PTEN*, mRNA expression (IlluminaHiSeq) in the TCGA breast invasive carcinoma (Pearson’s correlation (r) = 0.5504; *P* = 0.0001). **c** Graph showing the number of TNBC patients (TCGA) with T2 and above or < T2 in the low or high PTEN group. Statistical significance was determined by χ2 analysis. **P* < 0.05.  **d** Graph showing the number of TNBC patients (TCGA) with Stage II and above or Stage I in the low or high PTEN group. Statistical significance was determined by χ2 analysis. **P* < 0.05. **e** The scatter plot for the correlation of TNBC samples between PTEN, protein expression (RPPA) and AKT1_PT308, protein expression (RPPA) in the TCGA breast invasive carcinoma (Protein, RPPA) data (Pearson’s correlation (r) = -0.5478; *P* = 0.0001). **f** Heat-map showing 3,009 significantly different mRNAs between the high and low *PTEN* expressing TNBC samples obtained from the TCGA analysis. Red indicates up-regulation and blue for down-regulation. *n* = 10 per group.

**Figure S2. Workflow showing the whole genome siRNA screen in isogenic PTEN positive or negative TNBC cells.**

**a** Protein expressions of PTEN, phospho-AKT (p-AKT), AKT, phospho-ERK (p-ERK) and ERK expression in MCF10A, MDA-MB-468-TR-PTEN and MDA-MB-468-TR-EV with indicated treatments. GAPDH was used as a loading control. **b** A schematic diagram showing fluorescently labelling of MDA-MB-468-TR-PTEN and MDA-MB-468-TR-EV cells. Plasmids pCherryFP-N1 or p-EGFP-N1 were stably transfected into these two cell lines, respectively. Single clones were picked and sorted by fluorescence-activated cell sorting (FACS), and named as MDA-MB-468-TR-PTEN/CherryFP or MDA-MB-468-TR-EV/GFP. **c** A schematic diagram showing the whole genome siRNA screen in PTEN+ and PTEN- cell lines. DOX-treated MDA-MB-468-TR-PTEN/CherryFP (PTEN+) or MDA-MB-468-TR-EV/GFP (PTEN-) cells were mixed and transfected at a 1:1 ratio in 96-well plates. Cells were fixed with 4% paraformaldehyde at 96 h post transfection. Fluorescence was read on an EnVision 2102 Plate-reader.

**Figure S3. Candidate genes essential for the survival of PTEN-inactive TNBC cells are identified by a whole genome siRNA screen.**

**a** The response of cell lines to 21,121 siRNA pools in 3 replicate screens based on Z-scores was analysed by Pearson’s correlation. Individual dot indicates the pool of siRNA. Top and bottom panels show reproducibility analysis between the replicates in PTEN- cells and PTEN+ cells, respectively. **b** Heat-map showing 4,647 genes that have significant decrease in cell viability between PTEN+ and PTEN- TNBC cells obtained from the whole genome siRNA screen. Red indicates the high Z-scores and blue for low Z-scores. *n* = 3 per group.

**Figure S4. WDHD1 is required for the survival of PTEN null TNBC cells cultured in 2D.**

Protein expression of WDHD1 in HCC1806 (**a**), BT20 (**b**), MDA-MB-157 (**c**), MDA-MB-231 (**d**), MDA-MB-468 (**e**), HCC1395 (**f**), HCC1937 (**g**) and HCC38 (**h**) with indicated transfections in 2D cultures. β-tubulin was used as a loading control. Graphs showing relative cell viability in HCC1806 (**a**), BT20 (**b**), MDA-MB-157 (**c**), MDA-MB-231 (**d**), MDA-MB-468 (**e**), HCC1395 (**f**), HCC1937 (**g**) and HCC38 (**h**) with indicated transfections cultured in 2D cultures. Cell-Titer Glo® assay was performed to measure cell viability. Data are mean ± SEM. *n* = 3 per group. **P* < 0.05. ** *P* < 0.01.

**Figure S5.** **WDHD1 is required for the survival of PTEN null TNBC cells cultured in 3D.**

Representative phase contrast microscopy images of PTEN WT TNBC cell line BT20 (**a**) or MDA-MB-231 (**b**) with indicated transfections cultured in 3D. Scale bar: 50 µm. Graphs showing sphere formation efficiency, sphere volume and cell viability (Cell-Titer Glo® assay) in BT20 (**a**) or MDA-MB-231 (**b**) with indicated transfections cultured in 3D. Data are mean ± SEM. *n* = 3 samples per group. **P* < 0.05.

**Figure S6. TCGA analysis suggests an important role of WDHD1 in cell cycle regulation.**

(**a**) Heat-map showing differentially expressed genes (DEGs) in TNBC samples with the low *WDHD1* compared to those with the high WDHD1 obtained from the TCGA analysis. Red indicates up-regulation and blue for down-regulation. *n* = 10 per group. (**b**) Functional enrichment (ToppGene) of up-regulated DEGs in the high WDHD1 group was visualised on a bar chart, showing number of shared mRNAs (genes) and -Log10 (*P* value).

**Figure S7. Essential roles of WDHD1 in cell cycle in PTEN null TNBC cell lines.**

Protein expression of WDHD1 in MDA-MB-468 (**a**), HCC1395 (**b**), BT20 (**c**) and MDA-MB-231 (**d**) with indicated transfections. β-tubulin was used as a loading control. Representative flow cytometry histograms of percentage of cells in G1, S and G2/M phases of cell cycle from MDA-MB-468 (**a**) or HCC1395 (**b**) with indicated transfections. Graphs showing the percentage of cells in S-phase from MDA-MB-468 (**a**) or HCC1395 (**b**) with indicated transfections. In **c** and **d**, graphs showing the percentage of cells in G1, S or G2/M phases from BT20 (**c**) and MDA-MB-231 (**d**) with indicated transfections. Data are mean ± SEM. *n* = 3 samples per group. **P* < 0.05. ***P* < 0.01.

**Supplementary Tables**

**Table S1.** Expressions of 47 candidate mRNAs essential for the survival of PTEN-inactive TNBC cells in the TCGA samples with the high *vs.* low PTEN.

**Table S2.** 47 candidate genes essential for the survival of PTEN-inactive TNBC cells are identified by a whole genome siRNA screen.

**Table S3.** Functional enrichment (ToppGene) of WDHD1 binding partners identified via IP-MS.
