# Supplementary figures and images for: WDHD1 is essential for the survival of PTEN-inactive triple-negative breast cancer
Source: Cell Death Dis. 2020 Nov 21;11(11):1001. doi: 10.1038/s41419-020-03210-5 (PMC7680459; doi:10.1038/s41419-020-03210-5)

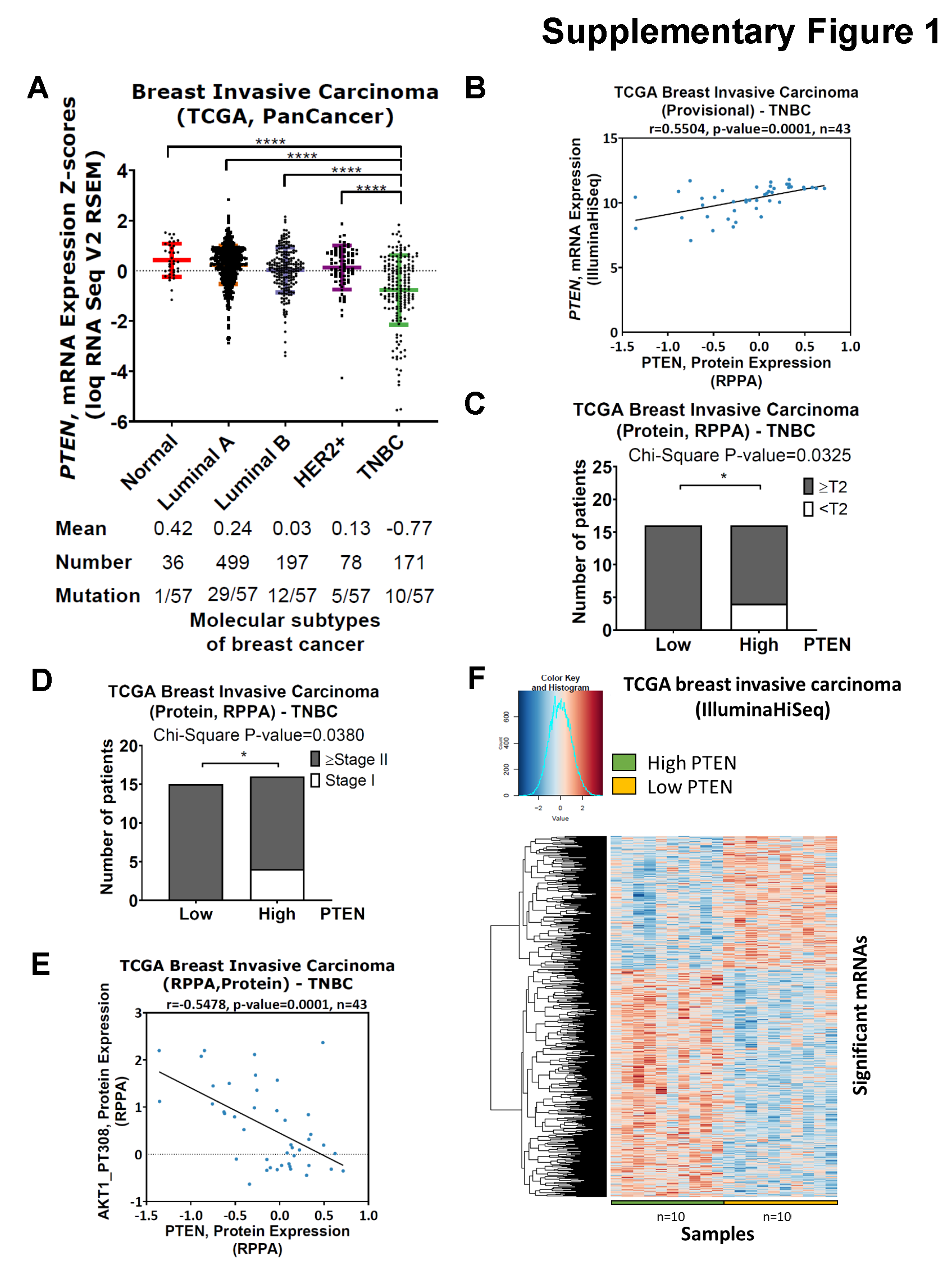

Supplement: Supplementary file 2 — Supplementary Figure 1 [file 41419_2020_3210_MOESM2_ESM.tif]

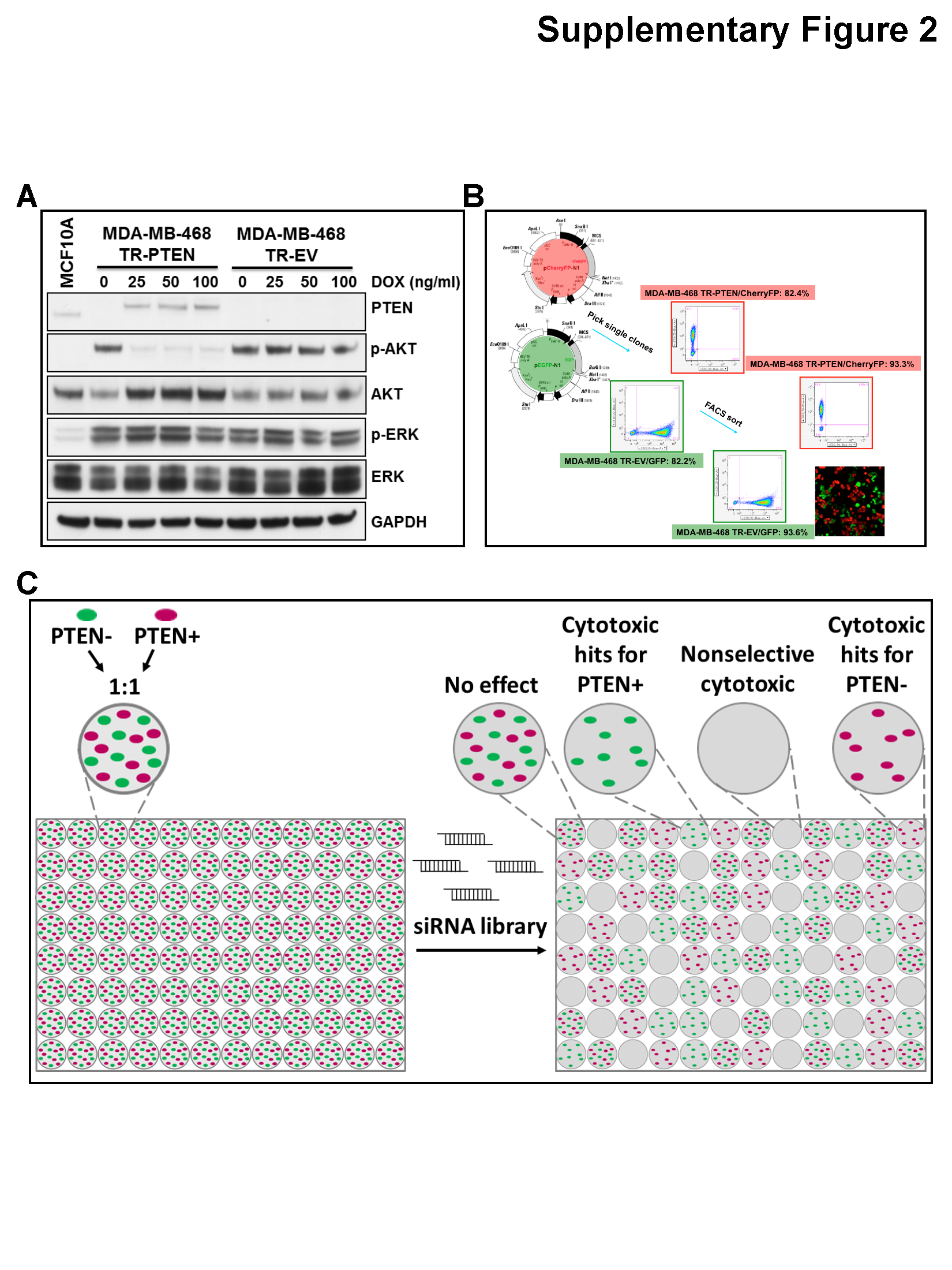

Supplement: Supplementary file 3 — Supplementary Figure 2 [file 41419_2020_3210_MOESM3_ESM.tif]

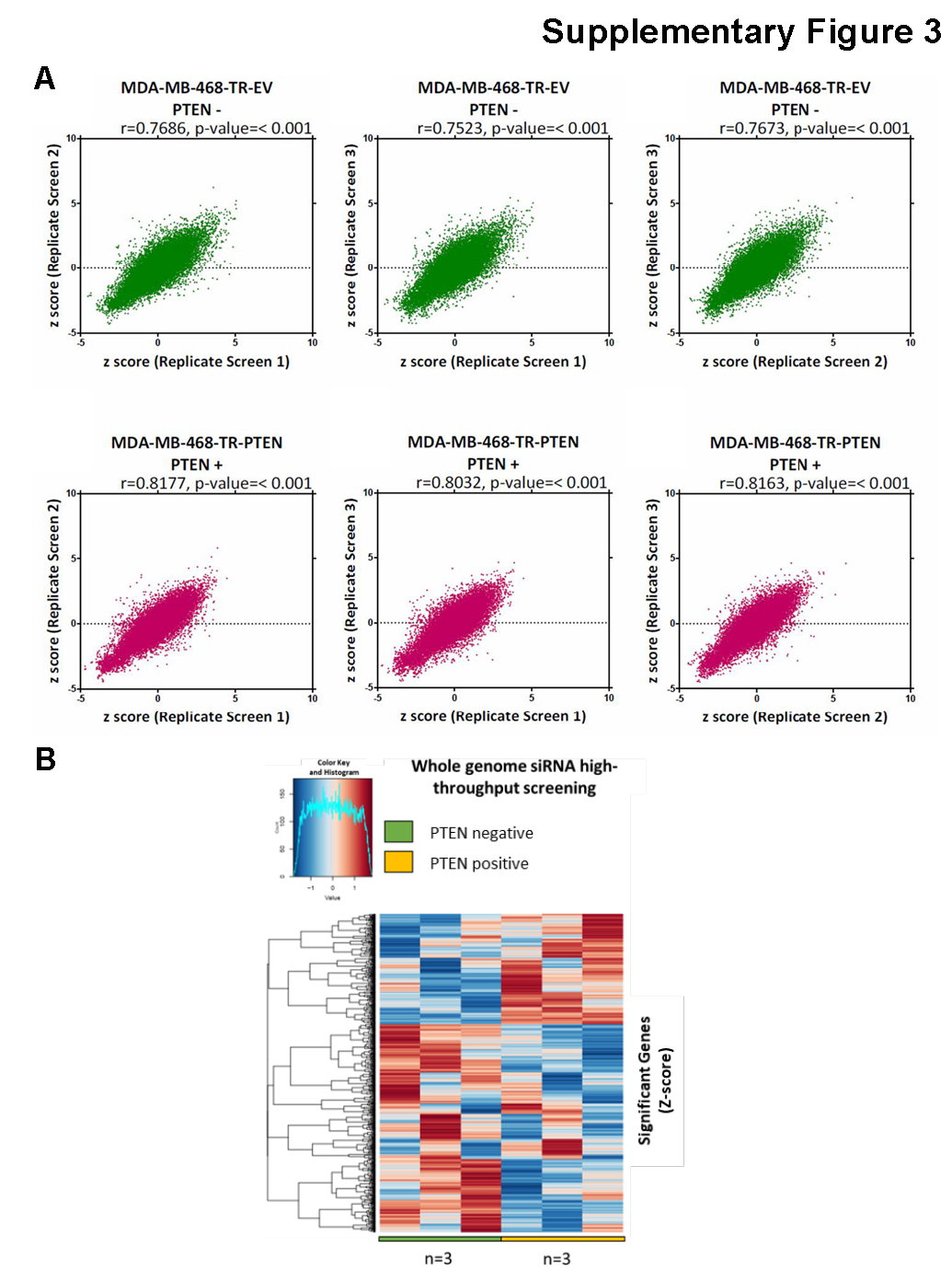

Supplement: Supplementary file 4 — Supplementary Figure 3 [file 41419_2020_3210_MOESM4_ESM.tif]

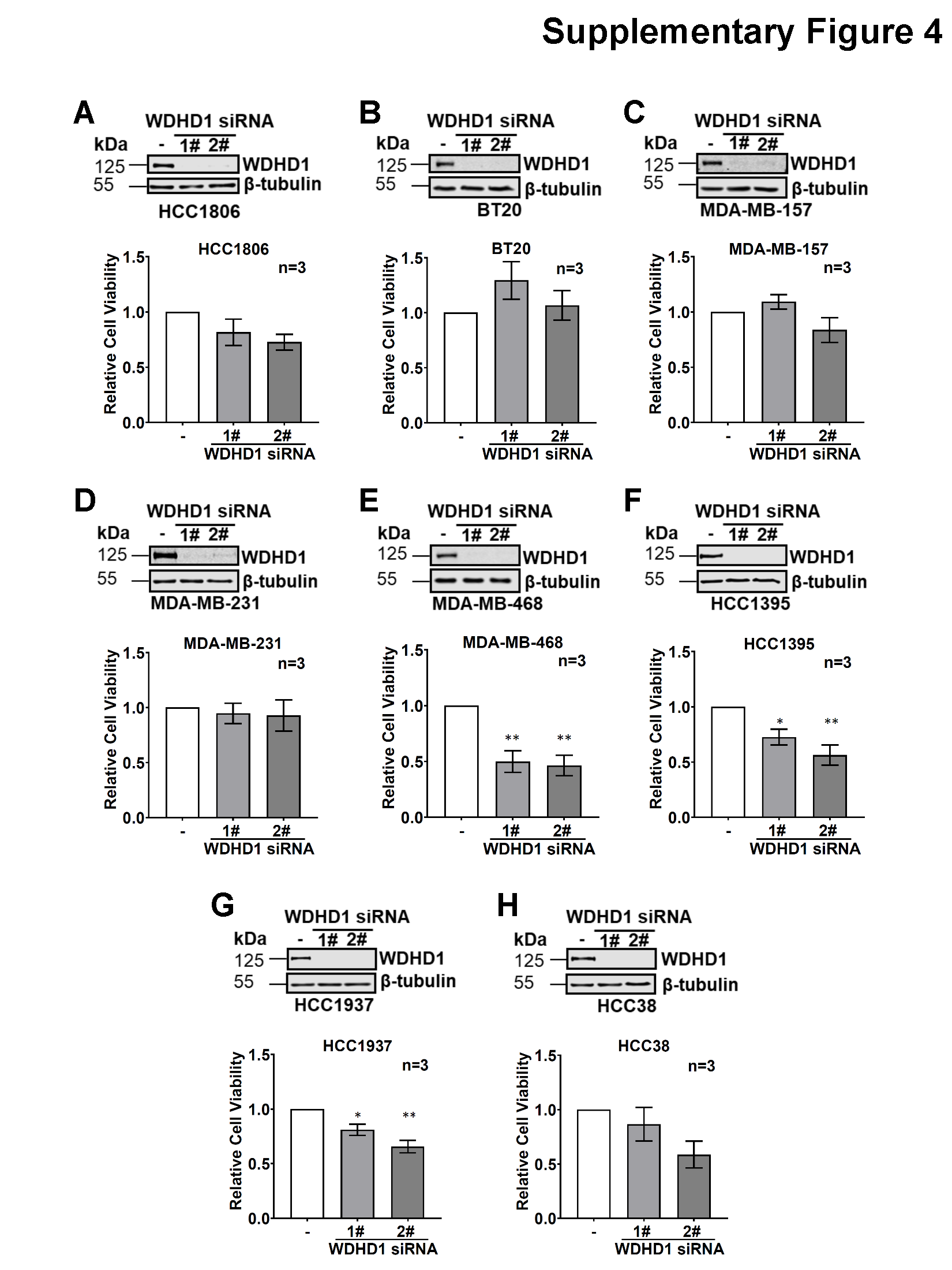

Supplement: Supplementary file 5 — Supplementary Figure 4 [file 41419_2020_3210_MOESM5_ESM.tif]

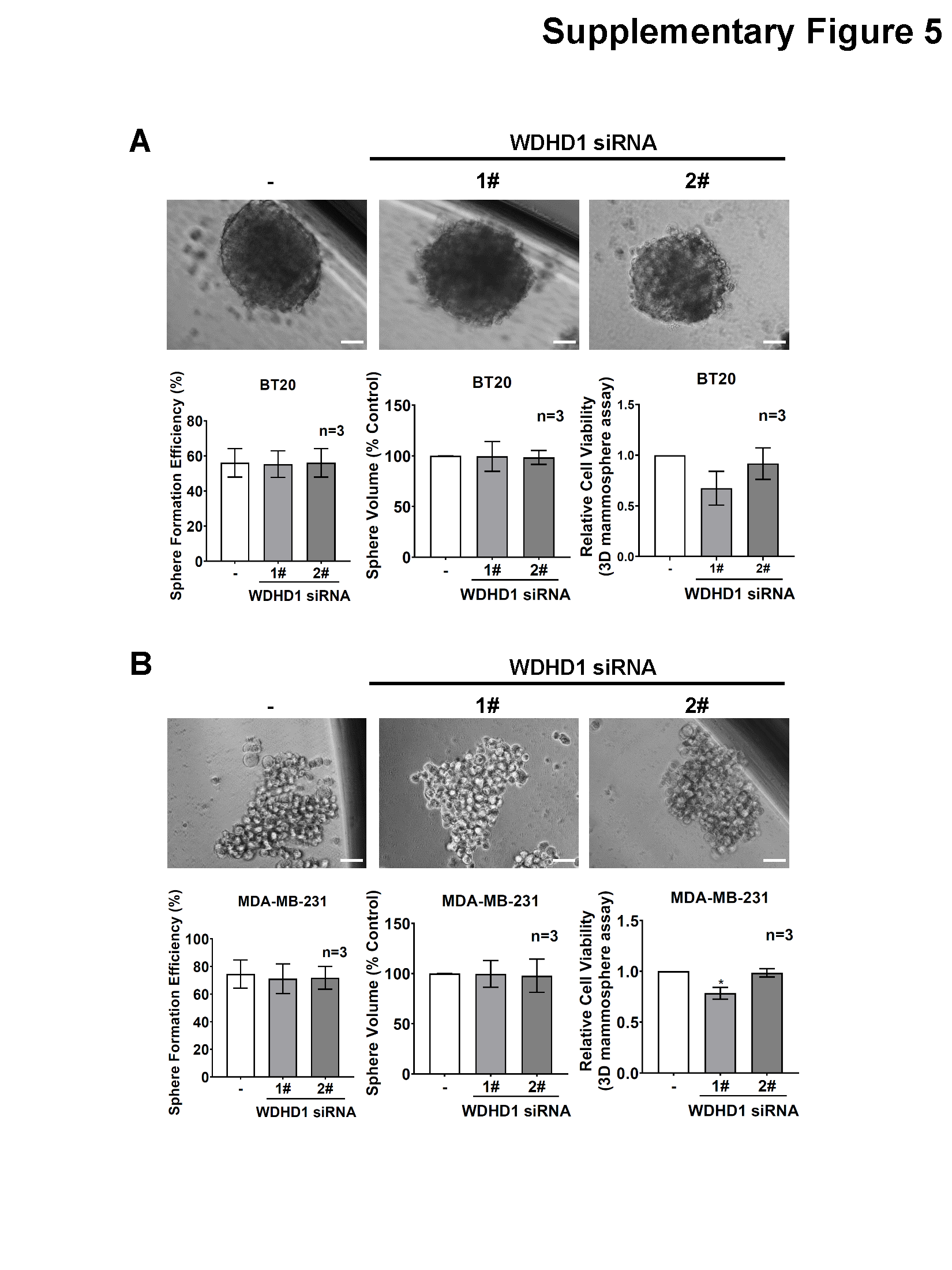

Supplement: Supplementary file 6 — Supplementary Figure 5 [file 41419_2020_3210_MOESM6_ESM.tif]

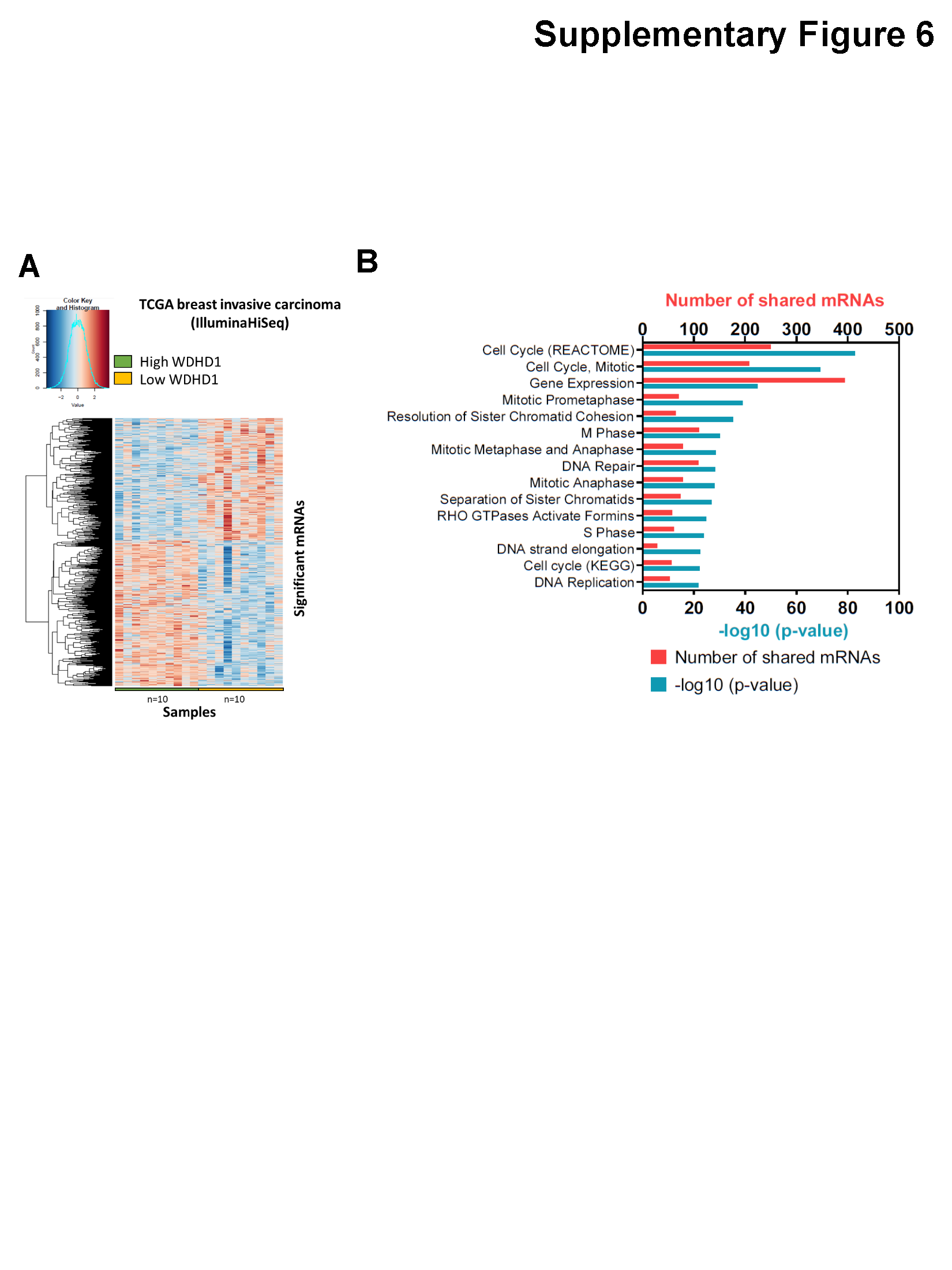

Supplement: Supplementary file 7 — Supplementary Figure 6 [file 41419_2020_3210_MOESM7_ESM.tif]

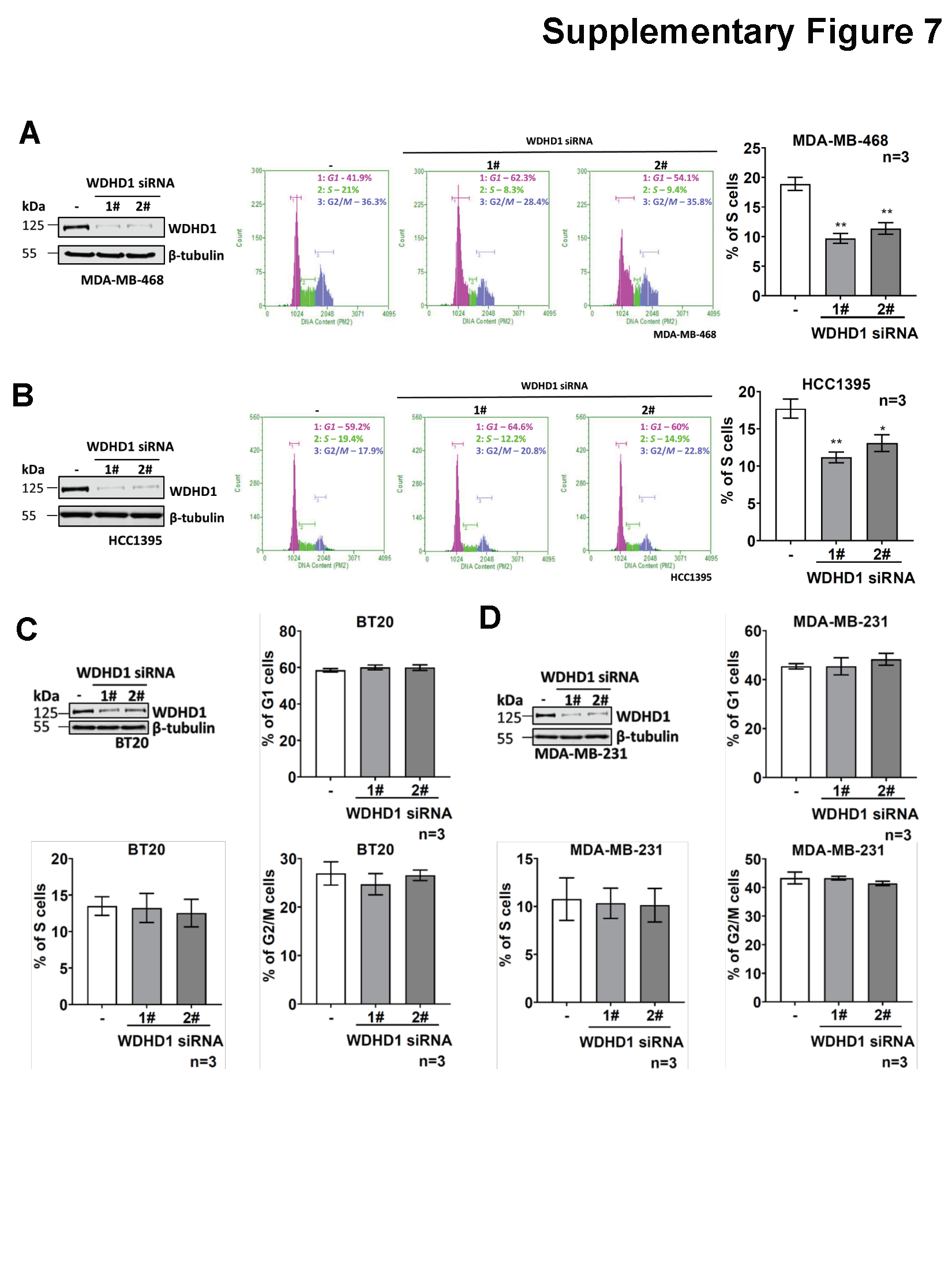

Supplement: Supplementary file 8 — Supplementary Figure 7 [file 41419_2020_3210_MOESM8_ESM.tif]
